# LncRNA like NMRK2 mRNA functions as a key molecular scaffold to enhance mitochondrial respiration of *NONO-TFE3* rearranged renal cell carcinoma in an NAD^+^ kinase-independent manner

**DOI:** 10.1186/s13046-023-02837-4

**Published:** 2023-09-28

**Authors:** Yi Chen, Yanwen Lu, Lei Yang, Wenliang Ma, Yuhan Dong, Shuoming Zhou, Ning Liu, Weidong Gan, Dongmei Li

**Affiliations:** 1 Immunology and Reproduction Biology Laboratory & State Key Laboratory of Analytical Chemistry for Life Science, Medical School, Nanjing University, Nanjing, Jiangsu 210093 China; 2 Jiangsu Key Laboratory of Molecular Medicine, Nanjing University, Nanjing, Jiangsu 210093 China; 3 Department of Urology, Affiliated Drum Tower Hospital of Medical School, Nanjing University, Nanjing, Jiangsu 210008 China; 4 Department of Clinical Biobank & Institute of Oncology, Affiliated Hospital of Nantong University, Nantong, Jiangsu 226000 China; 5 Department of Urology, Nanjing First Hospital, Nanjing Medical University, Nanjing, Jiangsu 210001 China

**Keywords:** *NONO-TFE3* rRCC, Mitochondrial respiration, Transcriptional-translational conflict, lncRNA like mRNA, NMRK2, NAD^+^ kinase-independent

## Abstract

**Background:**

*NONO-TFE3* rearranged renal cell carcinoma (*NONO-TFE3* rRCC) is one of a subtype of *TFE3* rRCCs with high malignancy and poor prognosis. Compared with clear cell RCC, *NONO-TFE3* rRCC shows a preference for mitochondrial respiration. We recently identified that the upregulation of nicotinamide ribokinase 2 (NMRK2) was associated with enhanced mitochondrial respiration and tumor progression in *TFE3* rRCC.

**Methods:**

A tumor-bearing mouse model was established to verify the pro-oncogenic effect of NMRK2 on *NONO-TFE3* rRCC. Then the expression of NMRK2 RNA and protein was detected in cell lines and patient specimens. The NMRK2 transcripts were Sanger-sequenced and blasted at NCBI website. We constructed dCas13b-HA system to investigate the factors binding with NMRK2 RNA. We also used molecular experiments like RIP-seq, IP-MS, FISH and fluorescence techniques to explore the mechanisms that long non-coding RNA (lncRNA) like NMRK2 mRNA promoted the mitochondrial respiration of *NONO-TFE3* rRCC. The efficacy of the combination of shRNA (NMRK2)-lentivirus and metformin on *NONO-TFE3* rRCC was assessed by CCK-8 assay.

**Results:**

In this study, we confirmed that NMRK2 showed transcriptional-translational conflict and functioned as lncRNA like mRNA in the *NONO-TFE3* rRCC. Furthermore, we revealed the molecular mechanism that NONO-TFE3 fusion suppressed the translation of NMRK2 mRNA. Most importantly, three major pathways were shown to explain the facilitation effects of lncRNA like NMRK2 mRNA on the mitochondrial respiration of *NONO-TFE3* rRCC in an NAD^+^ kinase-independent manner. Finally, the efficacy of combination of shRNA (NMRK2)-lentivirus and metformin on *NONO-TFE3* rRCC was demonstrated to be superior than either agent alone.

**Conclusions:**

Overall, our data comprehensively demonstrated the mechanisms for the enhanced mitochondrial respiration in *NONO-TFE3* rRCC and proposed lncRNA like NMRK2 mRNA as a therapy target for *NONO-TFE3* rRCC.

**Graphical Abstract:**

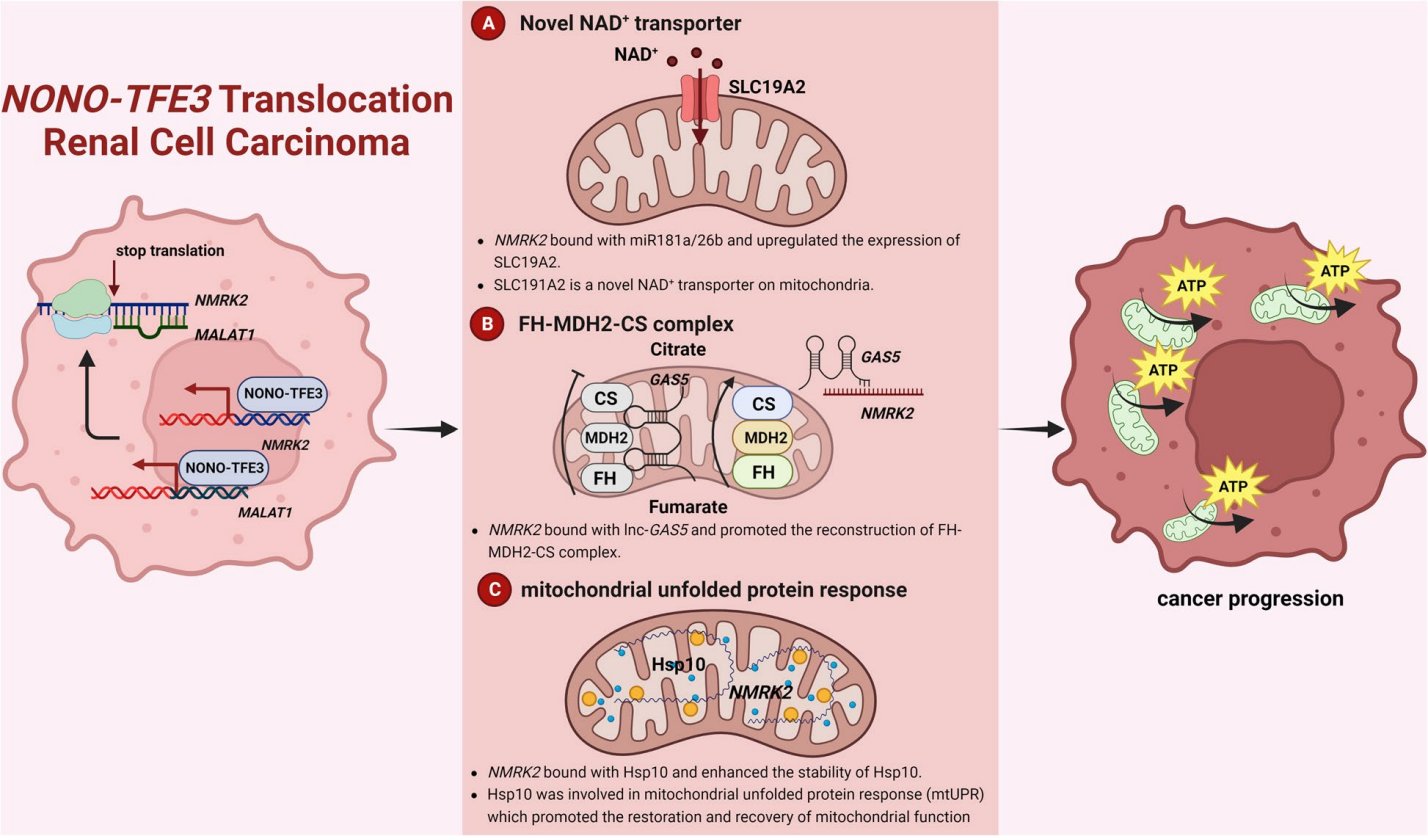

**Supplementary Information:**

The online version contains supplementary material available at 10.1186/s13046-023-02837-4.

## Background

*TFE3*-rearranged renal cell carcinoma (*TFE3* rRCC) is a member of MiT family translocation renal cell carcinomas (MiT tRCCs) which are more tumorigenic and metastatic than common RCCs [1–5]. *TFE3* rRCC is caused by a reciprocal translocation between *TFE3* on chromosome Xp11.2 and another chromosome, leading to the aberrant overexpression of active TFE3 fusion protein [6–10]. Wild-type TFE3 protein plays a central role in responding to internal and external environment changes related to metabolism regulation [11–13]. It was reported that the TFE3 protein could promote fatty acid catabolism [14] and mitochondrial function [15] to reduce the risk of obesity, implying that the TFE3 fusion protein could drive a unique energy metabolic signature in *TFE3* rRCC. Our previous study identifies that *TFE3* rRCC exerts a preference for oxidative phosphorylation, which is different from common RCCs [16, 17], and nicotinamide ribokinase 2 (NMRK2) upregulated by TFE3 fusion is associated with enhanced mitochondrial respiration and tumor progression in *TFE3* rRCC [16].

*NMRK2* is the gene encoding the NAD^+^ precursor (NMN) kinase [18, 19]. NAD^+^ is a crucial coenzyme involved in various redox reactions, including glucose metabolism, lipid metabolism, etc. [20]. Studies reported that the reduction of NAD^+^ grossly affects the expression of mitochondrial-related genes and impairs mitochondrial function [21, 22]. In our previous research, the high-level expression of NMRK2 in *TFE3* rRCC facilitates mitochondrial respiration and tumor progression [16]. However, it is proved that NMRK2 has a high transcriptional level but low translational level in *NONO-TFE3* rRCC, which is considered as transcriptional-translational conflict in recent research [23].

As one of the common subtypes of *TFE3* rRCC,* NONO-TFE3* rRCC results from the fusion between the *NONO* gene (1–9 exons; 1–11 exons) and *TFE3* gene (6–10 exons) [9, 24]. NONO protein is involved in nearly all phases of gene regulation [25], including pre-mRNA slicing [9, 26–28], RNA transport [29–31], and transcription regulation [32, 33]. The *NONO* part in the *NONO-TFE3* gene may endow this fusion protein with new functions. Recently, studies concerning *NONO-TFE3* rRCC indicate that this subtype of *TFE3* rRCC has a disturbed RNAs network, such as the downregulated lncRNA TRAF3IP2-AS1 [34, 35] and the upregulated circMET [36]. However, the reason why NMRK2 mRNA isn’t translated and the role of non-protein-translating, namely lncRNA like, NMRK2 mRNA in *NONO-TFE3* rRCC remains unexplored.

To address these questions, we conducted metabolic and non-coding RNA related studies using *TFE3* rRCC cell models and mouse models as well as the tumors from *TFE3* rRCC patients. Our findings revealed that NONO fragment in the NONO-TFE3 fusion protein disturbed the movement of ribosomes on NMRK2 mRNA, which led to the translation suppression of NMRK2 mRNA in *NONO-TFE3* rRCC. Further, we verified that lncRNA like NMRK2 mRNA worked as a molecular scaffold by binding with miR26b/miR181a, lnc-GAS5, or Hsp10 protein to promote the mitochondrial respiration of *NONO-TFE3* rRCC. Our studies clarified the critical role of lncRNA like NMRK2 mRNA in the metabolic adaption of *NONO-TFE3* rRCC in an NAD^+^ kinase-independent manner and underlined the importance of RNA therapeutics.

## Materials and methods

All the essential resources are shown in sTable 1.

### Mouse model

Six-week-old BALB/c nude female mice were used in this subcutaneous tumor xenograft model. Firstly, HK-2 cells were transfected with pCDH-DsRed/pCDH-NONO-TFE3, shRNA (NC)/shRNA (NMRK2). Following transfection, cells were selected with puromycin until all the non-transfected cells died. When the cells reached enough, 1 × 10^6^ cells in each group were suspended with 50 μL DMEM and 50 μL matrigel and then injected subcutaneously (100 μL /per mouse) in the axillary posterior of the forelimbs. After 4 weeks, animals were sacrificed, and the subcutaneous tumor masses were taken out for subsequent studies, including hematoxylin and eosin (HE) staining and immunohistochemical (IHC). All procedures were approved by the Animal Care and use Committee of Nanjing University under the animal protocol number SYXK (Su) 2009–0017.

### Plasmid models

The CDS sequences of NONO-TFE3, NONO (1-9), TFE3 (6-10), FH, CS, MDH2, SLC19A2, HSPE1 and the promoter sequences of lnc-MALAT1 were obtained from Sanger sequencing or NCBI, and the 3’-UTR sequences of SLC19A2 were acquired from NCBI. Then the template sequences were amplified with Phanta Max Master Mix in UOK109 cells and subcloned into linearized plasmids pCDH-DsRed, pcDNA3.1-3xFlag, pBiFC-VC155, pBiFC-VN173, pCDH-EGFP, pGL3-Basic, and pmiRGLO with a ClonExpress II One Step Cloning Kit, respectively. The recombinant plasmids were transformed into Escherichia coli Stbl3 (pCDH-NT) or Escherichia coli DH5α (NONO-Flag, TFE3-Flag, NT-Flag, MDH2-Flag, VC-FH, VC-CS, VN-MDH2, pCDH-SLC19A2, pCDH-HSPE1, lnc-MALAT1-promoter, and SLC19A2-3’UTR) and grown in Luria broth (LB) agar plates supplemented with 0.1% ampicillin. After 16–24 h, colony PCR was used to check for recombinant plasmids in colonies. The positive colonies were further expanded in the LB medium with 0.1% ampicillin. Eventually, recombinant plasmids were extracted with EndoFree Mini Plasmid Kit. Mut Express II Fast Mutagenesis Kit was used to construct binding sites mutation or deletion plasmids of SLC19A2 3’-UTR.

The shRNA sequences targeting NMRK2, lnc-MALAT1, HSPE1, and SLC19A2 were obtained from Sigma websites and modified according to specific restriction sites. Then these sequences were inserted into linearized plasmid pLV-shRNA with a ligation mix. The recombinant plasmids were transformed into Escherichia coli DH5α and screened, expanded, and extracted as described before.

The primers for plasmid construction are shown in sTable 2.

### Lentivirus models

Lentivirus packaging was performed using HEK293T cells. 5 μg constructed shRNA or pCDH-DsRed was co-transfected with lentivirus packaging plasmids psPAX2 (4 μg) and pMD2G (2 μg) using LipoFiter 3.0 at a cell density of ~ 50% (take 10 mm dish as an example). After 6 h of transfection, the supernatant of the cell cultures was removed, and 10 mL of fresh DMEM medium was added. During the 48- and 72-h periods, virus supernatants were collected twice and filtered with a 0.45 μm filter membrane.

### Cell culture and transfection

The human renal clear cell carcinoma cell line 786-O, 769-P, ACHN, human kidney cortex/proximal tubule cell line HK-2, human embryonic kidney cell line HEK293T cells, and *TFE3* rRCC cell lines UOK120 and UOK109 were cultured in DMEM containing 10% FBS and 1% puromycin at 37 °C with 5% CO_2_. To assess mitochondrial function, glucose was replaced by galactose in the DMEM.

Plasmid transfection was performed using LipoFiter 3.0 according to the manufacturer’s protocol. Cells were examined for related RNA expression after 48 h of transfection. Lentivirus transfection was performed by virus supernatants and polybrene (8 μg/mL). After 72 h, cells were treated with puromycin to select infected cells. Then, related RNA was detected to determine transfection efficiency.

### Protein extraction and western blotting

After treatment, the total protein of cells was extracted with RIPA lysis with protease and phosphatase inhibitor cocktail on ice. BCA Protein Quantification Kit assessed protein concentration, then all protein concentrations in each group were adjusted to the lowest concentration using ddH_2_O. The protein was added with a 5 × loading buffer and boiled for 5 min.

A standard protocol was followed for electrophoresis, transfer to PVDF membranes, and blotting of proteins. Then the bands were incubated with specific primary antibodies, including NMRK2, TFE3, β-actin, Flag, SLC19A2, GAPDH, Tom20, MDH2, CS, FH, Bax, Caspase 3, Cleaved Caspase 3 and Hsp10 for a whole night at 4 °C. Afterward, at room temperature, anti-rabbit or anti-mouse secondary antibodies were incubated for 1 h. Finally, the protein expression was examined with FDbio-Femto ECL Kit and calculated with Image J. We used β-actin as the internal reference. The relative expression of the target protein was calculated as the grey value of the target protein bands/the grey value of the β-actin protein bands.

### RNA isolation and real-time quantitative PRC (q-PCR)

After treatment, the total RNA of cells was extracted with RNA Easy Isolation Reagent according to the manufacturer’s protocol, and the RNA concentration in each group was detected with Nanodrop. The RNA was reversed transcribed with HiScript 1st Strand cDNA Synthesis Kit. The q-PCR was performed using the SYBR Green Q-PCR Kit. We used the CT value of 18S rRNA as the internal reference, and the relative RNA expression was analyzed with the ΔΔCt method. The primers for RNAs are listed in sTable 2.

### Immunohistochemistry analysis (IHC)

The tumors from model mice or *TFE3* rRCC patients were embedded in paraffin and sectioned. Then the tissue sections were dewaxed with xylene, dehydrated with ethyl alcohol, antigen repaired with citric repair solution, and blocked with 3% BSA. The sections were incubated with specific primary antibodies, including NMRK2, Ki67, and TFE3, for the whole night in the humidified box at 4 °C. An IHC Kit was used for color development. Nuclear counterstaining was performed with hematoxylin. After completing the staining, the sections were dehydrated and sealed with neutral resin. Finally, the expression of the target protein was observed under a microscope and analyzed with Image J software.

### Hematoxylin and Eosin (HE) staining

The hematoxylin was added to the dewaxed and dehydrated sections for 5 min for nuclear counterstaining. Then the sections were performed with 1% hydrochloric alcohol for differentiation. An appropriate amount of eosin was added to the sections for 30 s. Then the sections were dehydrated again and sealed with neutral resin. Finally, the organizational structure of each section was observed under a microscope.

### ATP production assay

UOK109 cells with different treatment were cultured in a 6-well plate (3 × 10^5^ cells/per well) for 24 h. After treatment, the production of ATP in each group was detected by an Enhanced ATP Assay Kit and GloMax96 according to the manufacturer’s protocol.

### Cell counting kit-8 (CCK-8) assay

UOK109 cells transfected with shRNA (NC) or shRNA (NMRK2) or treated with metformin were cultured in 96-well plates (3 × 10^3^ cells/per well). At the corresponding time point, the cell viability of each group was detected by a Cell Counting Kit and Molecular Devices M3 according to the manufacturer’s protocol.

### Detection of mitochondrial function

The Seahorse XF Cell Mito Test Kit was used to carry out the mitochondrial function analysis of UOK109 cells with different treatments. Before the formal experiments began, UOK109 cells with different treatments were cultured in a Seahorse XFe96 cell culture microplate at the density of 1 × 10^4^ cells/ 80 μL/well. The Seahorse XF96 Analyzer was turned on to warm in advance. The probe plate was incubated overnight at 37 °C in an O_2_-free environment with 200 μL ddH_2_O. On the day of the experiment, the probe plate was secondary hydration with a hydration solution. The cell culture medium was replaced with an XF base medium containing 1 mM pyruvate, 2 mM glutamine, and 25 mM glucose, and the cells were placed into a 37 °C O_2_-free incubator for 1 h. The process of drug dispensing (1.5 μM oligomycin, 0.5 μM rotenone/antimycin A, and 1 μM FCCP) and addition should be completed within 45 min. As soon as the probe calibration was completed (15 min), the probe plate was replaced with the cell plate, and the detection began. The mitochondrial function was assessed with the OCR value.

### RNA fluorescence in situ hybridization (RNA FISH)

A RNA FISH Kit was used in this experiment. The dewaxing and dehydrating were performed for paraffin sections as described in IHC. Then, the proteinase K was used for 20 min at 37 °C. The sections were blocked with blocking solutions and added with a denaturation buffer for 8 min at 78 °C for denaturation. NMRK2, lnc-GAS5, 18S rRNA, and U6 probe were diluted according to the manufacturer’s protocol. The sections were incubated with probe working solutions overnight at 37 °C molecular hybridization. After incubation, the sections were washed at 60 °C and 37 °C for 3 times, respectively. Finally, the nuclei were stained with DAPI, and the sections were observed under a confocal microscopy. 4% paraformaldehyde was used to fix the cells, and methanol was performed for permeabilization. The remaining steps were the same as those stated above. The sequences for probes are shown in sTable 2.

### RNA Immunoprecipitation (RIP)

A RIP Kit was used in this experiment. Firstly, UOK109 cells with dCas13b-HA and sg-Con/sg-NMRK2 were collected by centrifugation at 1500 rpm for 5 min at 4 °C. The supernatant was discarded, and the cells were re-suspended in an equal pellet volume of complete RIP lysis buffer. The magnetic beads were prepared for immunoprecipitation by incubating with HA-antibody for 30 min at room temperature and then washed twice. Then the magnetic beads were suspended with 900 μL RIP immunoprecipitation buffer and incubated with 100 μL RIP lysate by rotating at 4 °C overnight. After incubation, the supernatant was discarded, and the beads were washed for six times. Finally, the binding RNA was purified by proteinase K and extracted with phenol chloroform. The primers for sg-RNAs are shown in sTable 2.

### Ribosomes purification

A ribosome Extraction Kit was used to purify the ribosomes in UOK109 with shRNA (NC) and shRNA (MALAT1). Firstly, 1–2 × 10^7^ treated UOK109 cells were collected and centrifuged at 500 × g for 5 min at 4 °C. They were incubated with 500–1000 μL ice-cold reagent A for 10 min and then were homogenized using a Dounce tissue grinder for 30–40 strokes on ice. The homogenates were centrifuged at 1000 × g for 5 min at 4 °C, and the supernatants were collected. After centrifuging them at 20000 × g for 10 min at 4 °C, the supernatants were collected again. The supernatants were centrifugation at 100,000 × g for 60 min at 4 °C, and the precipitates were collected. 400 μL ice-cold reagent B was added to the precipitates in each group, and the mixtures were centrifuged at 100000 × g for 60 min at 4 °C. The precipitates were purified ribosomes.

### Dual-Luciferase reporter assay

The reporter gene plasmids of MALAT1-promoter, SLC19A2-3’UTR, mutated SLC19A2-3’UTR and deleted SLC19A2-3’UTR were constructed and transfected into UOK109 or HEK293T as described before. For internal normalization, we used the Renilla luciferase gene plasmid pRL-TK. For SLC19A2-3’UTR, mutated SLC19A2-3’UTR and deleted SLC19A2-3’UTR, miR-26b or miR-181 mimic or negative control miRNA was co-transfected with them into HEK293T. A Dual-Luciferase Reporter Assay Kit was used to detect the relative fluorescence intensity in each group.

### Immunofluorescence

UOK109 cells (~ 3 × 10^4^) with different treatments (with no treatment, transfected with pLV-mitoDsRed, dcas13b-HA, and sg-NMRK2, transfected with pLV-mitoDsRed, VN-MDH2, and VC-CS/VC-FH) were cultured in 35 mm glass bottom dishes. For UOK109 cells with no treatment and transfected with pLV-mitoDsRed, dcas13b-HA, and sg-NMRK2, 4% paraformaldehyde was used to fix the cells for 5 min. Subsequently, the cells were perforated with 0.1% Triton X-100 and blocked with 3% bovine serum albumin (BSA). The cells were incubated with primary antibodies (Tom20 and SLC19A2) overnight. After the cells had been rinsed in PBS, the primary antibodies were visualized with fluorescent-tagged secondary antibodies. The nuclei were labeled with DAPI. For cells transfected with pLV-mitoDsRed, VN-MDH2, and VC-CS/VC-FH, the punch and antibody incubation steps were omitted. The primers for sg-RNAs are shown in sTable 2.

### NAD^+^/NADH assay

An NAD^+^/NADH Assay Kit was used to measure the NAD^+^ content in UOK109 cells with different treatments. 1 × 10^6^ UOK109 cells with different treatment were prepared and lysed with 200 μL NAD^+^/NADH extraction solution. Half of the lysis was taken into a 60 °C water bath for 30 min for NADH measurement, and the other half was for the total content of NAD^+^ and NADH. The working solutions were prepared by diluting stock solutions with ethanol and added to each sample well. After incubation for 10 min, color development solutions were added. The absorbance was measured at 450 nm.

### Mitochondria purification

UOK109, UOK120, or 786-O cells (~ 1 × 10^7^) were cultured in a 15 cm dish, and the mitochondria inside the cells were extracted and purified with a Cell Mitochondria Isolation Kit according to the manufacturer’s protocol. As described, the total mitochondrial protein and RNA were extracted with RIPA lysis and RNA Easy Isolation Reagent.

### Immunoprecipitation (IP)

The MDH2-Flag plasmid was transfected into UOK109 cells with shRNA (NC) or shRNA (NMRK2) as described before. All the groups were treated with no glucose medium for 24 h. Using the Anti-Flag Affinity Gel incubated overnight, the proteins binding with MDH2-Flag were immunoprecipitated. The expression of CS and FH in the binding protein mixture was detected by Western Blotting as described before.

### Cell apoptosis assay

UOK109 cells (5 × 10^5^ cells) with shRNA (NC), shRNA (HSPE1), and shRNA (NMRK2) were collected with pancreatin without EDTA and washed twice by PBS. Then cells were stained with 5 μL Annexin V-PE and 7-AAD for 10 min. The apoptosis rate of each group was detected by BD FACSCalibur flow cytometer.

### Mitochondrial membrane potential assay

UOK109 cells (3 × 10^4^ cells/per well) with shRNA (NC), shRNA (NMRK2), shRNA (HSPE1) or shRNA (NMRK2) + pCDH-HSPE1 were cultured in 35 mm glass bottom dishes. Positive control was prepared by being treated with CCCP (10 μM) for 30 min. After that, all the cells were treated with 1 mL TRME staining working solution for 30 min. After incubation, the culture fluid was removed, and the plate was washed twice with the medium. The mitochondrial membrane potential was detected under fluorescence confocal microscopy.

## Results

### *NMRK2* facilitated the mitochondrial respiration and tumor progression of *NONO-TFE3* rRCC

**Fig. 1:**
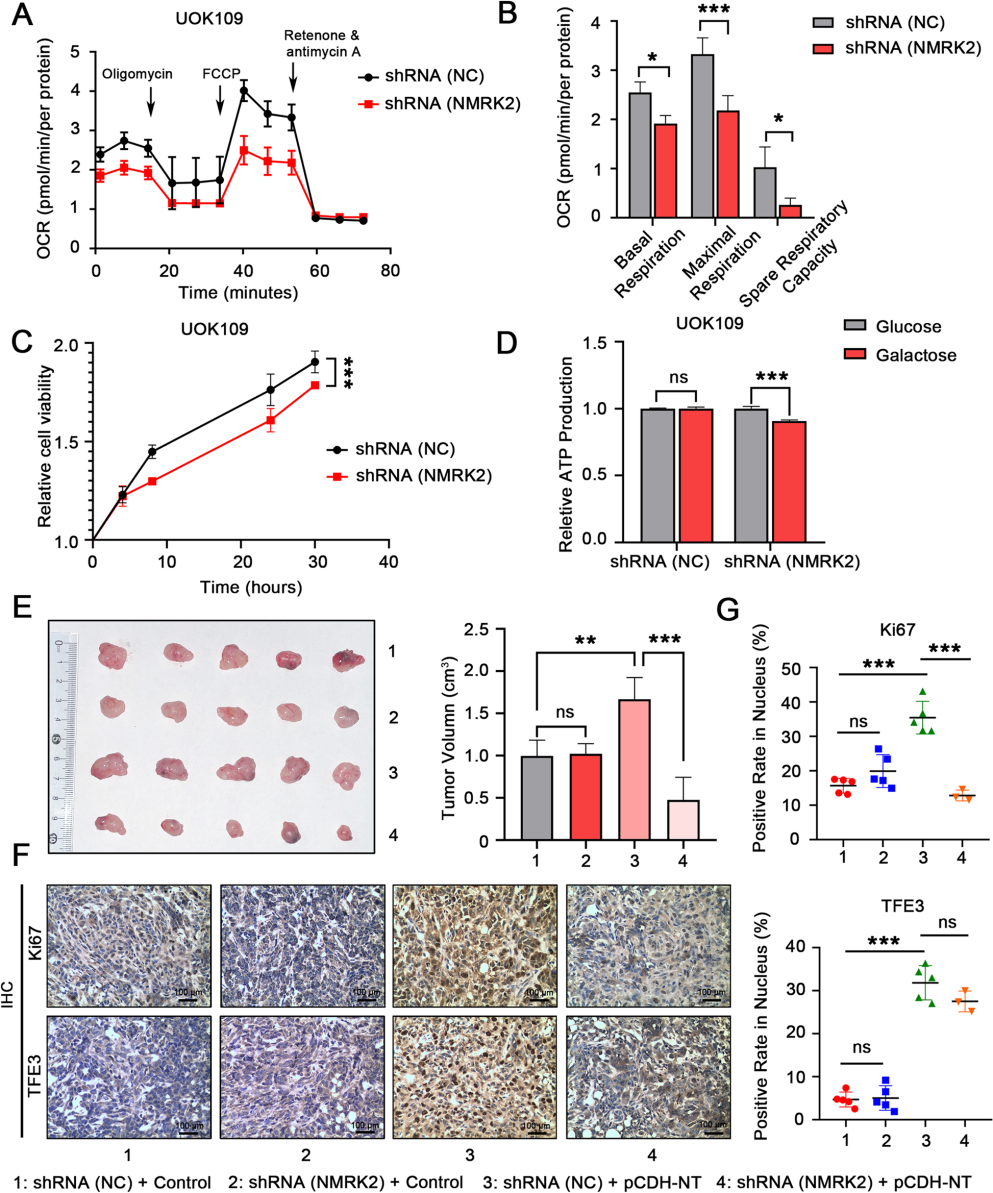
*NMRK2* promoted mitochondrial respiration and tumor growth of *NONO-TFE3* rRCC. **A** UOK109 cells were transfected with lentivirus shRNA (NC) or shRNA (NMRK2). The mitochondrial respiration of cells in each group was measured by the Seahorse XF Extracellular Flux Analyzer with a Seahorse XF Cell Mito Stress Kit, **B** and analyzed with GraphPad Prism 8. **C** And the cell proliferation viability was analyzed with a CCK-8 assay kit. **D** UOK109 cells were transfected with lentivirus shRNA (NC) or shRNA (NMRK2) and cultured in a medium with glucose or galactose. The ATP production was detected with an ATP assay kit. **E** HK-2 cells were transfected with lentivirus pCDH-DsRed/pCDH-NT, shRNA (NC)/shRNA (NMRK2). Twenty female nude mice were randomly divided into 4 groups with 5 mice per group. Then 1 × 10^6^ transfected HK-2 cells were injected subcutaneously in mice. After 4 weeks, the tumors from each group were removed and measured. **F** The expression of Ki67 and TFE3 protein of the tumors from each group was detected by IHC. **G** The statistic results of Ki67 and TFE3 were calculated with Image J and GraphPad Prism 8. Data are presented as the mean ± SEM. *P < 0.05, **P < 0.01, ***P < 0.001

Our previous studies have demonstrated that *NMRK2* is highly expressed in *TFE3* rRCC, leading to the preference for mitochondrial respiration [16]. To probe the role of *NMRK2* on *NONO-TFE3* rRCC, mitochondrial function was detected by measuring the oxygen consumption rate (OCR) with the Seahorse XF Cell Mito Stress Kit. The result showed that knock-down of *NMRK2* could impair the mitochondrial respiration of UOK109 cells (Fig. 1A, B). CCK-8 assay indicated that the knock-down of *NMRK2* inhibited UOK109 cells viability (Fig. 1C). Additionally, in UOK109 cells, the ATP production ability, as well as number and morphology of mitochondria, were impaired when NMRK2 was knocked down (sFig. 1A, B). Then, we substituted galactose for glucose to inhibit glycolysis activity to evaluate the effect of *NMRK2* on mitochondrial respiration. The result showed that *NMRK2* interference decreased the efficiency of ATP production in UOK109 cells (Fig. 1D). Further, a tumor-bearing mouse model was used to verify the reduction in tumor volume when *NMRK2* was knocked down in group pCDH-NT (Fig. 1E). The protein expression of Ki67 was also decreased after the knock-down of *NMRK2* in group pCDH-NT (Fig. 1F, G). In summary, increased expression of *NMRK2* enhanced the mitochondrial respiration of *NONO-TFE3* rRCC.

### NMRK2 showed transcriptional-translational conflict and functioned as lncRNA like mRNA in *NONO-TFE3* rRCC

**Fig. 2:**
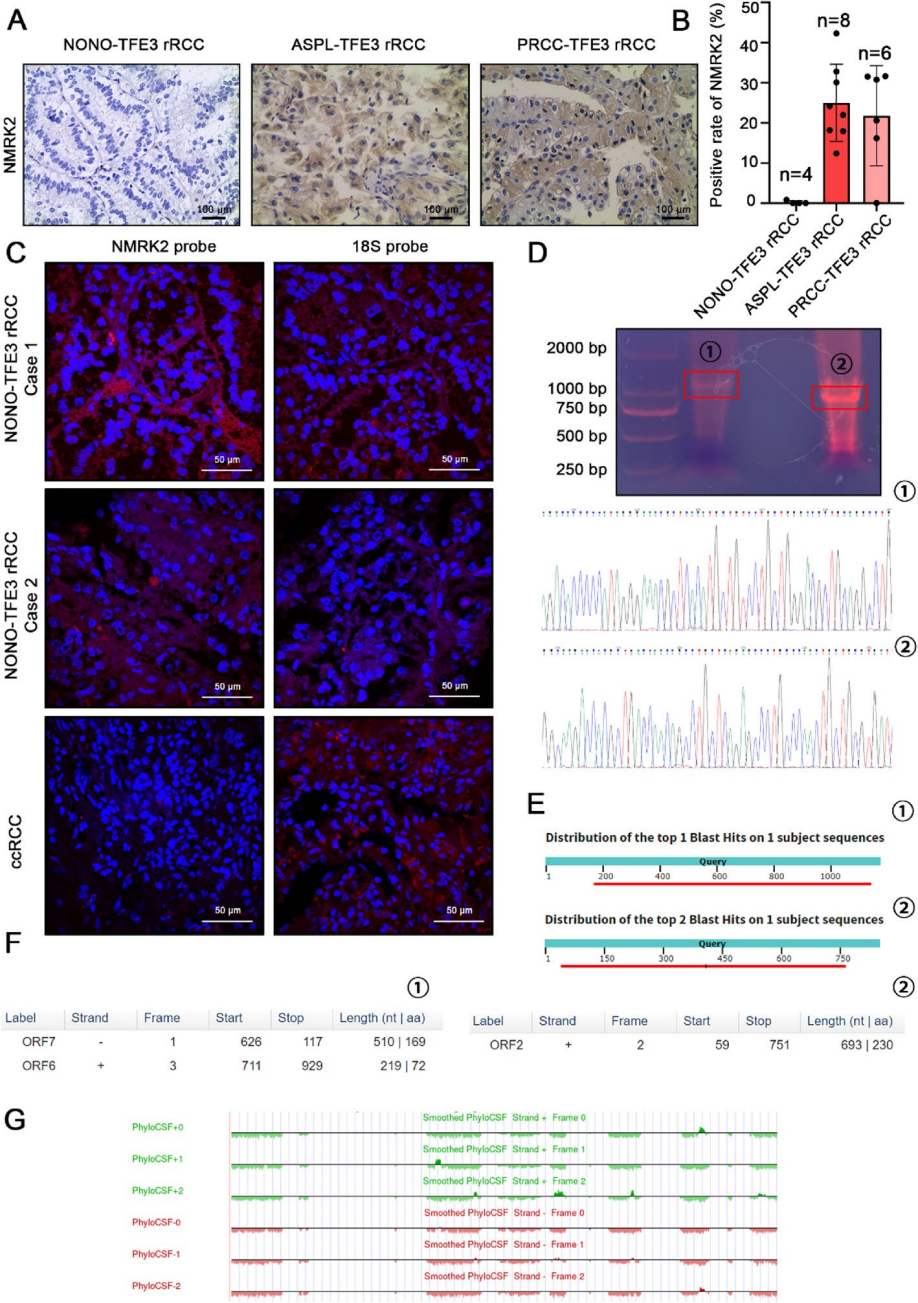
NMRK2 had transcriptional-translational conflict process and functioned as lncRNA like mRNA in *NONO-TFE3* rRCC. **A**, **B** The expression of NMRK2 protein in *NONO-TFE3* rRCC, *ASPL-TFE3* rRCC, or *PRCC-TFE3* rRCC was detected by IHC and calculated by GraphPad Prism 8. **C** The expression of NMRK2 RNA in *NONO-TFE3* trRCC and ccRCC was detected by the FISH assay. **D** The longer and shorter transcripts of NMRK2 in UOK109 cells were amplified by PCR and Sanger-sequenced. **E** The sequence alignment between NMRK2 transcripts in UOK109 cells and NMRK2 in Genbank database were performed at NCBI website. **F** The potential ORFs of NMRK2 transcripts in UOK109 cells were predicted with ORFfinder viewer. **G** The phyloCSF of NMRK2 were calculated at UCSC website

To compare the expression of NMRK2 protein between different subtypes of *TFE3* rRCC, a total of 40 *TFE3* rRCC cases was detected using IHC [16]. Among these cases, 8/8 *ASPL-TFE3* rRCC cases and 5/6 *PRCC-TFE3* rRCC cases showed solid or moderate positivity for NMRK2 staining. In contrast, 4/4 *NONO-TFE3* rRCC patients showed negativity for NMRK2 staining (Fig. 2A, B). Furthermore, consistent with previous results [16], the FISH result showed a high transcript level of NMRK2 in *NONO-TFE3* rRCC, which was considered as transcriptional-translational conflict (Fig. 2C). To probe whether the sequence of NMRK2 was changed in *NONO-TFE3* rRCC, the longer and shorter transcripts were amplified by PCR and Sanger-sequenced (Fig. 2D). Sequence alignments were conducted using BLAST at NCBI website. Except for the 100 bp bases at 5’-end and 3’-end of DNAs which were prone to sequencing errors, the sequences of NMRK2 transcripts in UOK109 cells were consistent with the Genbank database (Fig. 2E). In addition, NMRK2 transcripts in UOK109 cells have predictive open reading frames (ORFs) longer than 200 nt (Fig. 2F). The codon substitution frequency of NMRK2 transcripts were accessed by phyloCSF at UCSC website, and the results showed that the NMRK2 transcripts in UOK109 cells were conserved (Fig. 2G). In all, non-protein-translating NMRK2 mRNA with ORFs in *NONO-TFE3* rRCC was not lncRNA in the strict sense of the word, but it might function as lncRNA like mRNA. Thus, we referred the NMRK2 in *NONO-TFE3* rRCC as lncRNA like mRNA.

### The translation of NMRK2 protein was suppressed by NONO fragment of NONO-TFE3 fusion protein

**Fig. 3:**
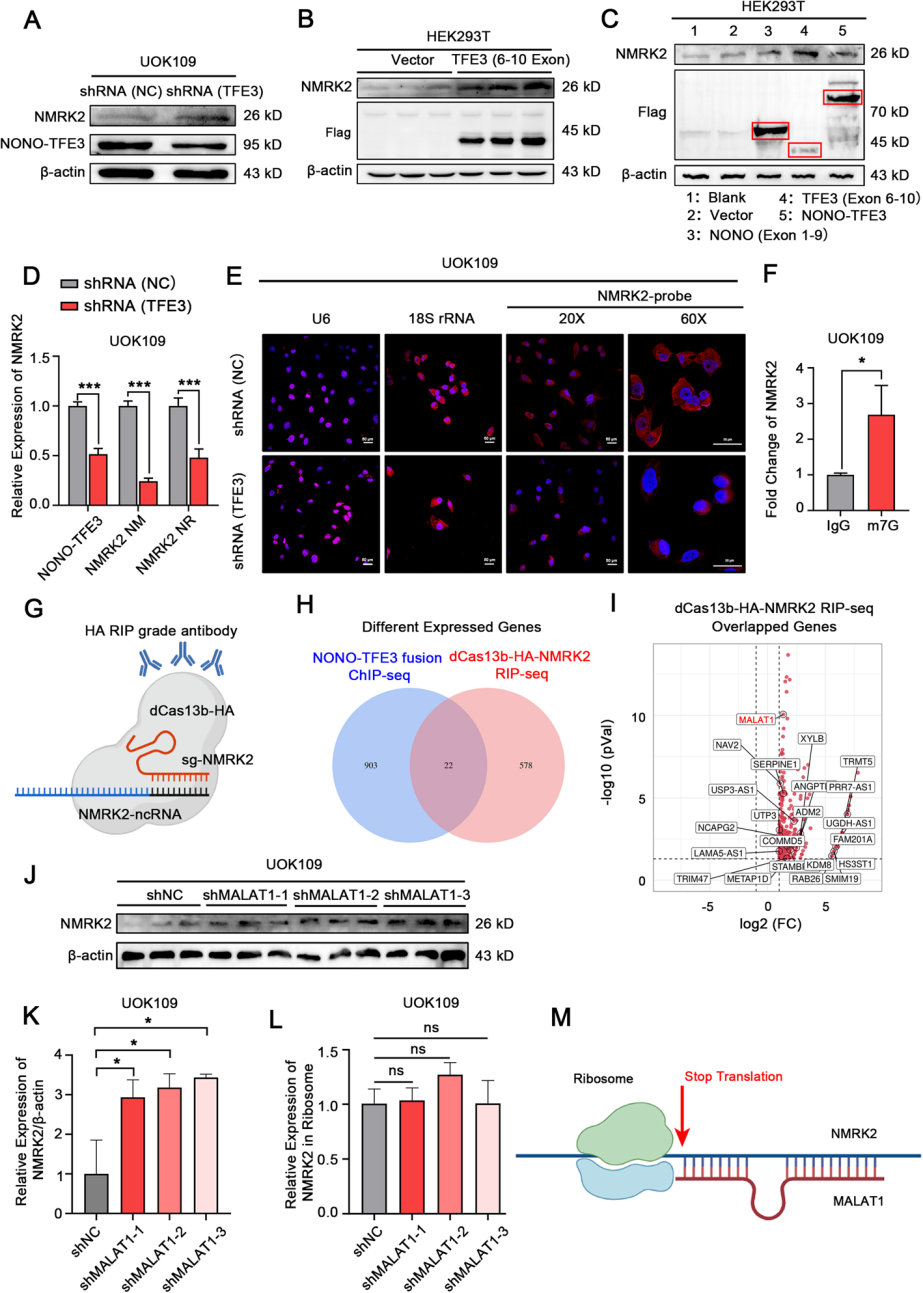
The NMRK2 protein expression was suppressed by NONO fragment of NONO-TFE3 fusion in *NONO-TFE3* rRCC. **A** UOK109 cells were transfected with lentivirus shRNA (NC) or shRNA (TFE3). The expression of NMRK2 protein was measured by Western Blotting. β-actin was used as the internal reference. **B** HEK293T cells were transfected with vector plasmid or TFE3 (6–10 exon)-Flag plasmid, and the NMRK2 protein expression was measured by Western Blotting. β-actin was used as the internal reference. **C** HEK293T cells were transfected with vector, NONO (1–9 exon)-Flag, TFE3 (6–10 exon)-Flag, or NONO-TFE3-Flag plasmid, and the NMRK2 protein expression was measured by Western Blotting. β-actin was used as the internal reference. **D** UOK109 cells were transfected with lentivirus shRNA (NC) or shRNA (TFE3). The expression of NMRK2 NM or NMRK2 NR was measured by qPCR. 18S rRNA was used as the reference gene. **E** The subcellular distribution of NMRK2 mRNA was detected with the FISH assay. The U6 probe and 18S rRNA probe were used for positive control for the nucleus and cytoplasm locations, respectively. **F** The enrichment of m7G in NMRK2 mRNA was assessed by RIP assay. Rabbit IgG was used as a negative control. **G** A schematic representation of dCas13b-HA RIP assay. **H** The Wayne diagram showed the intersection of the RIP-seq dataset and the NONO-TFE3 fusion protein ChIP-seq dataset. **I** The volcano map showed the overlapped genes between the RIP-seq dataset and the NONO-TFE3 fusion protein ChIP-seq dataset. **J** UOK109 cells were transfected with Lentivirus shRNA (NC), shRNA (MALAT1)-1, shRNA (MALAT1)-2, or shRNA (MALAT1)-3. The NMRK2 protein expression was detected by Western Blotting. β-actin was used as the internal reference. **K** And the results were quantified with Image J. **L** The ribosomes in UOK109 cells from each group were extracted and purified with a Ribosome Extraction Kit. The total RNA in ribosomes was isolated with RNA Easy Isolation Reagent, and the expression of NMRK2 mRNA was measured by qPCR. 18S rRNA was used as the reference gene. **M** A schematic representation of the translation repression machinery by lnc-MALAT1. Data are presented as the mean ± SEM. *P < 0.05, ***P < 0.001

Since we have confirmed that NONO-TFE3 fusion protein transcriptionally up-regulated NMRK2 in our previous study [16], we next further explore the mechanism of translation inhibition of NMRK2 in *NONO-TFE3* rRCC. Interestingly, NMRK2 protein began to be up-regulated once NONO-TFE3 fusion was knocked down (Fig. 3A). To explore which fragment of NONO-TFE3 fusion prevents the expression of NMRK2 protein, HEK293T cells were transfected with the NONO-TFE3-Flag plasmid, NONO (Exon 1–9)-Flag plasmid or TFE3 (Exon 6–10)-Flag plasmid, respectively. The result of Western Blotting showed that TFE3 fragment promoted the expression of NMRK2 protein, while NONO fragment suppressed the enhancing function of TFE3 fragment on NMRK2 protein level (Fig. 3B, C).

To explore the mechanism that the expression of NMRK2 protein was inhibited by NONO-TFE3 protein, specific primers were designed to target the protein-coding transcripts (NM) and non-protein-coding transcripts (NR) of NMRK2. The result showed that both the NM and NR of NMRK2 were positively transcriptionally regulated by NONO-TFE3 fusion (Fig. 3D). On the other hand, most of the NMRK2 mRNA was located in the cytoplasm (Fig. 3E). And there was the modification of cap-m7G which guided the ribosomes to recognize mRNAs (Fig. 3F), suggesting that NONO-TFE3 protein did not affect the maturation processing and nuclear transport of NMRK2 mRNA.

To further explore the molecular mechanism of NONO-TFE3 protein suppressing the translation of NMRK2 mRNA, UOK109 cells were transfected with dcas13b-HA and sgRNA targeting NMRK2 mRNA. Then RIP-seq was used to enrich and detect the binding RNA of NMRK2 mRNA (Fig. 3G). The intersection of the RIP-seq dataset and NONO-TFE3 fusion protein ChIP-seq dataset [37] was used for further analysis. The result showed that the lnc-MALAT1 was the gene with the greatest change (Fig. 3H, I). We first verified the transcriptionally regulatory relationship between lnc-MALAT1 and NONO-TFE3 fusion. And the results of q-PCR and luciferase indicated that NONO-TFE3 fusion positively regulated the expression of lnc-MALAT1 (sFig. 2A, B). However, PRCC-TFE3 fusion exerted no significant regulatory effect on lnc-MALAT1 (sFig. 2C, D), which could explain the differential expression of NMRK2 protein in *NONO-TFE3* rRCC and *PRCC-TFE3* rRCC. Lnc-MALAT1 knockdown in UOK109 promoted the translation of NMRK2 (Fig. 3J, K). To determine whether lnc-MALAT1 affected the binding between NMRK2 mRNA and ribosomes, the ribosomal RNA was extracted in group shRNA (NC) or shRNA (MALAT1). The result demonstrated that the amount of NMRK2 mRNA bound to ribosomes showed no significant differences before or after the knockdown of lnc-MALAT1 (Fig. 3L). Therefore, these results demonstrated that lnc-MALAT1 upregulated by NONO fragment of NONO-TFE3 fusion prevented ribosomes from scanning the NMRK2 mRNA and interrupted the translation of NMRK2 by binding to NMRK2 mRNA (Fig. 3M).

### LncRNA like NMRK2 mRNA promoted mitochondrial respiration by up-regulating the mitochondrial NAD^+^ transporter SLC19A2

**Fig. 4:**
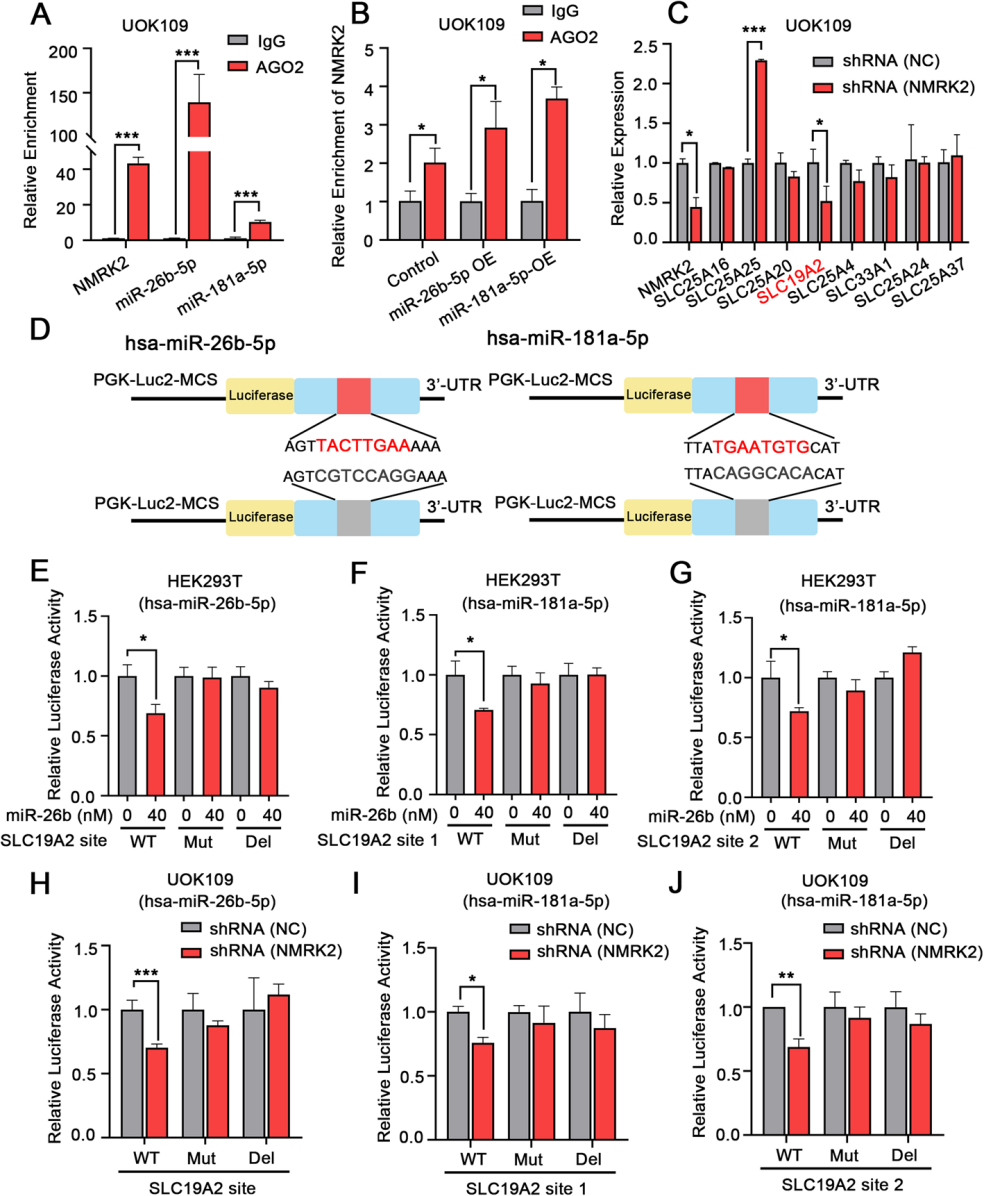
LncRNA like NMRK2 mRNA upregulated the expression of SLC19A2 by adsorbing miR-26b and miR-181a. **A** The enrichment of AGO2 on NMRK2, miR-26b, or miR-181a was assessed by RIP assay. Rabbit IgG was used as a negative control. **B** UOK109 cells were transfected with control, miR-26b or miR-181a mimic RNA. The enrichment of AGO2 on NMRK2 was assessed by RIP assay. Rabbit IgG was used as a negative control. **C** UOK109 cells were transfected with lentivirus shRNA (NC) or shRNA (NMRK2). The expression of the target genes of miR-26b and miR-181a were detected by q-PCR. 18S rRNA was used as the reference gene. **D** Schematic of binding sites mutation or deletion between the 3’-UTR of SLC19A2 and miR-26b/miR-181a. **E**–**G** HEK293T cells were co-transfected with the reporter plasmids containing the wild, mutated, or deleted binding sites between the 3’-UTR of SLC19A2 and miR-26b/miR-181a and 0 nM or 40 nM miR-26b/miR-181a mimic RNA. The luciferase activity in each group was measured with a Dual-Luciferase Reporter Assay Kit. **H**-**J** UOK109 cells were transfected with lentivirus shRNA (NC) or shRNA (NMRK2) and reporter plasmids containing the wild, mutated, or deleted binding sites between the 3’-UTR of SLC19A2 and miR-26b/miR-181a. The luciferase activity in each group was measured with a Dual-Luciferase Reporter Assay Kit. Data are presented as the mean ± SEM. *P < 0.05, **P < 0.01, ***P < 0.001

Since lncRNA like NMRK2 mRNA was mainly distributed in the cell cytoplasm, we hypothesized that NMRK2 might function as a molecular sponge to modulate miRNAs. By going through the RNAInter database, miR-26b and miR-181a were predicted to be the potential targets of lncRNA like NMRK2 mRNA. To verify the binding of NMRK2 with miR-26b/miR-181a, an AGO2-RIP assay was performed. Results showed that the AGO2 antibody could pull down both NMRK2 and miR-26b/miR-181a (Fig. 4A). When miR-26b or miR-181a was overexpressed, more NMRK2 was enriched by the AGO2 antibody, which further validated their binding potential (Fig. 4B). Using the miRbase database and miRDB database, some of the solute carrier family members were found to be the potential targets of miR-26b/miR-181a. Among them, SLC19A2 was markedly downregulated after the knockdown of NMRK2 (Fig. 4C, sFig. 3A). Also, the expression of SLC19A2 was sharply decreased after the over-expression of miR-26b/miR-181a mimic RNAs (sFig. 3B, C). Therefore, SLC19A2 was selected as a putative target of miR-26b/miR-181a for further observation. The potential binding sites of miR-26b or miR-181a on the 3’-UTR of SLC19A2 were predicted by the miRDB database. We mutated or deleted the binding targets, respectively (Fig. 4D). Luciferase assay showed that the luciferase activity of the SLC19A2 reporter with wild-type binding sites could be decreased, but not the reporter plasmids with mutation or deletion of the binding sites, which confirmed that miR-26b/miR-181a exerted a regulatory role on SLC19A2 expression (Fig. 4E, F, G). Furthermore, the luciferase activity of the wild-type SLC19A2 reporter, but not the mutant or deleted SLC19A2 reporter, could be decreased when NMRK2 was knocked down (Fig. 4H, I, J). In all, lncRNA like NMRK2 mRNA could positively regulate the expression of SLC19A2 by competitively binding to miR-26b/miR-181a.

**Fig. 5:**
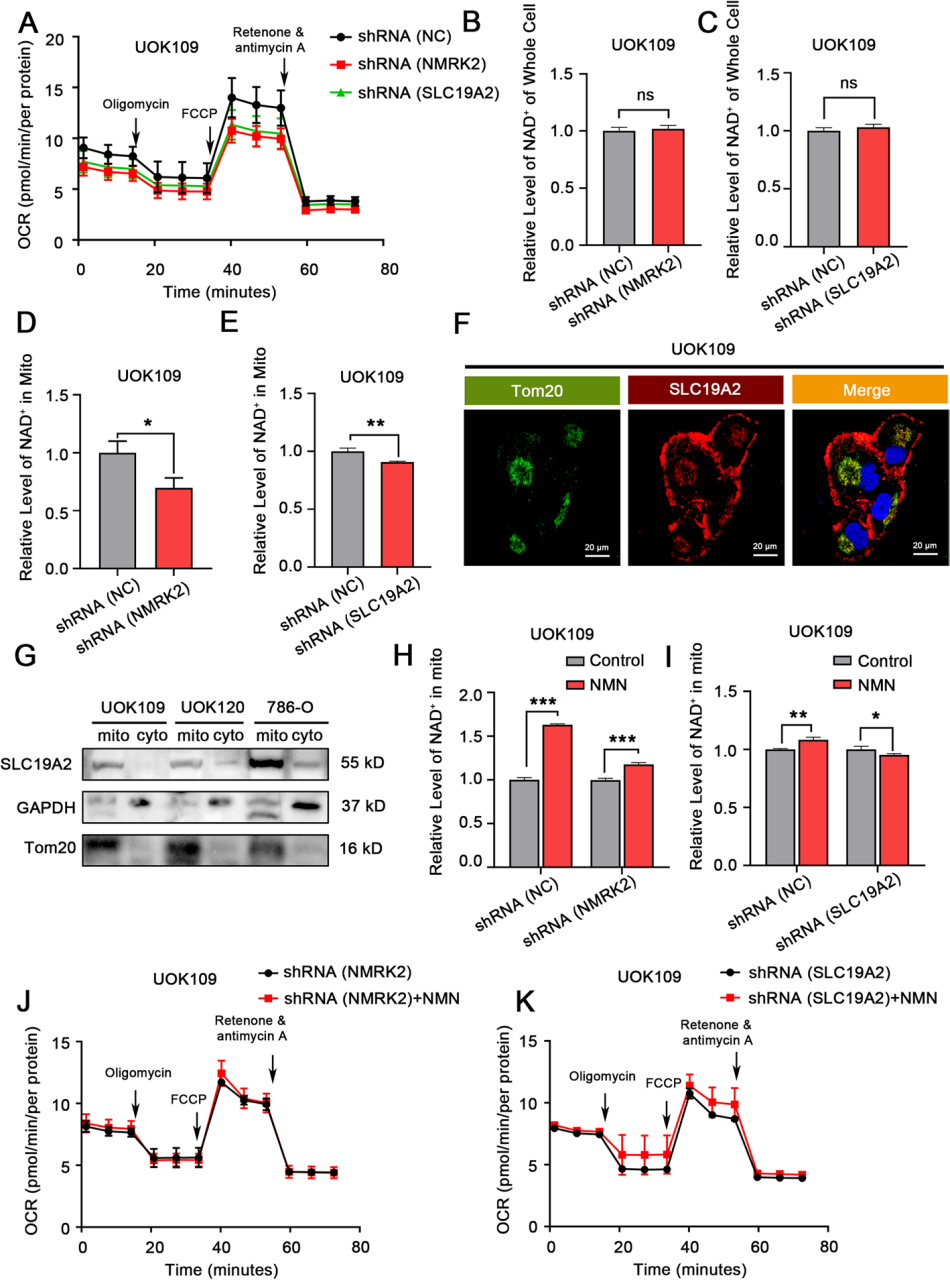
SLC19A2 promoted the mitochondrial respiration of *NONO-TFE3* rRCC by increasing the NAD^+^ transportation into mitochondria. **A** UOK109 cells were transfected with lentivirus shRNA (NC), shRNA (NMRK2), or shRNA (SLC19A2). The mitochondrial respiration of cells in each group was measured by the Seahorse XF Extracellular Flux Analyzer with a Seahorse XF Cell Mito Stress Kit. **B**, **C** And the NAD^+^ level of the whole cell or **D**, **E** of mitochondria of cells in each group was measured with an NAD^+^/NADH Assay Kit. **F** The subcellular localization of the SLC19A2 protein in UOK109 cells was observed by IF. **G** The mitochondria in UOK109, UOK120, and 786-O cells were isolated, and the total protein in the cytoplasm and mitochondria was extracted. The expression of SLC19A2 protein in cytoplasm and mitochondria was detected by Western Blotting. GAPDH and Tom20 were used as the positive control for periplasmic protein and mitochondrial protein, respectively. **H**, **I** UOK109 cells were transfected with lentivirus shRNA (NC), shRNA (NMRK2), or shRNA (SLC19A2). Then the cells in each group were treated or untreated with 100 μM NMN for 24 h. The NAD^+^ level in mitochondria was measured with an NAD.^+^/NADH Assay Kit. **J**, **K** And the mitochondrial respiration of cells in each group was measured by the Seahorse XF Extracellular Flux Analyzer with a Seahorse XF Cell Mito Stress Kit. Data are presented as the mean ± SEM. *P < 0.05, **P < 0.01, ***P < 0.001

The result of the Seahorse XF Cell Mito Stress test demonstrated that the knock-down of NMRK2 or SLC19A2 could impair the mitochondrial respiration of UOK109 cells (Fig. 5A, sFig. 4A). To explore how SLC19A2 promotes the mitochondrial respiration of *NONO-TFE3* rRCC, NAD^+^ content was measured using an NAD^+^/NADH assay kit when NMRK2 or SLC19A2 was knocked down. The result demonstrated that the total amount of NAD^+^ had no decreasing trend before or after the knockdown of NMRK2 or SLC19A2 (Fig. 5B, C). However, the knockdown of NMRK2 or SLC19A2 caused the reduction of mitochondrial NAD^+^ (Fig. 5D, E). As the NAD^+^ synthesis occurs only in the cytosol, we speculated that SLC19A2 could be a novel NAD^+^ transporter to mitochondria. Subcellular localization of SLC19A2 showed that SLC19A2 was expressed on the cell and mitochondria membranes (Fig. 5F, G), which was the premise of transporting NAD^+^ into mitochondria. After supplementation of the NAD^+^ precursor NMN, the change in mitochondrial NAD^+^ level in group shRNA (NMRK2) or shRNA (SLC19A2) was inferior to that in group shRNA (NC) (Fig. 5H, I). Also, there was no significant difference in the recovery of the respiration flux after the knockdown of NMRK2 or SLC19A2 when supplemented with NMN (Fig. 5J, K, sFig. 4C, D). The phenomenon above could be reversed when SlC19A2 was overexpressed in group shRNA (NMRK2) (sFig. 4B, E–G). In summary, by adsorbing miR26b/miR181a, lncRNA like NMRK2 mRNA up-regulated SLC19A2 which was an NAD^+^ transporter to promote the mitochondrial respiration of *NONO-TFE3* rRCC.

### LncRNA like NMRK2 mRNA promoted the mitochondrial respiration by relieving the inhibitory effect of lnc-GAS5 on the TCA cycle

**Fig. 6:**
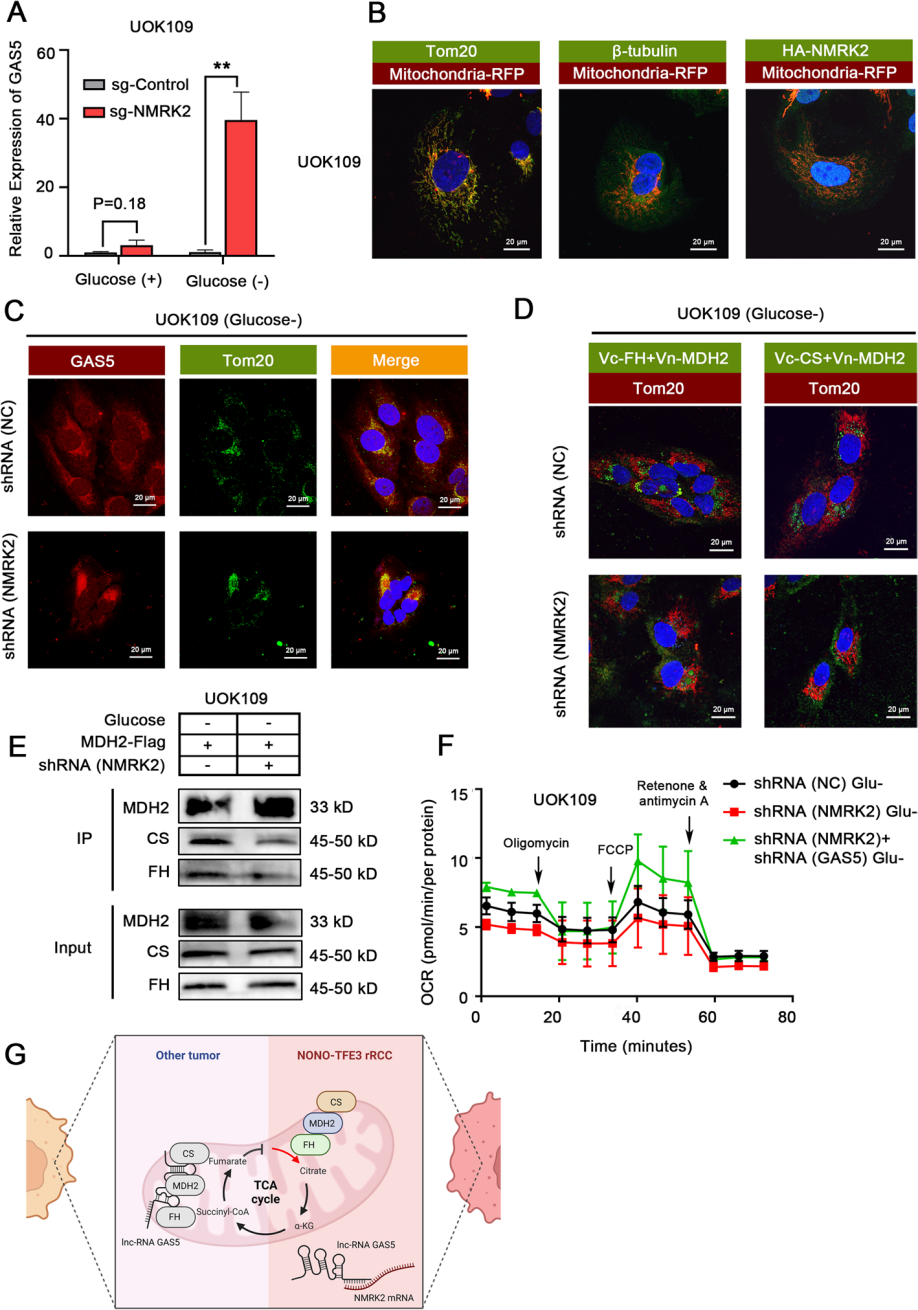
LncRNA like NMRK2 mRNA promoted the mitochondrial respiration of *NONO-TFE3* rRCC by relieving the inhibitory effect of lnc-GAS5 on the TCA cycle. **A** UOK109 cells were co-transfected with dCas13b-HA and sg-Control/sg-NMRK2. The enrichment of lnc-GAS5 on NMRK2 under glucose deprivation for 24 h was assessed by RIP assay. Rabbit IgG was used as a negative control. **B** UOK109 cells were co-transfected with dCas13b-HA, sg-NMRK2, and mitochondria-RFP. The subcellular localization of lncRNA like NMRK2 mRNA was observed by the IF assay. Tom20 and β-tubulin were used as the positive and negative control for mitochondrial protein. **C** UOK109 cells were transfected with lentivirus shRNA (NC) or shRNA (NMRK2) and cultured with glucose deprivation for 24 h. The subcellular localization of lnc-GAS5 was observed by the FISH assay. **D** UOK109 cells were co-transfected with V_N_-MDH2 and V_C_-FH/V_C_-CS and cultured with glucose deprivation for 24 h. The binding between MDH2, FH, and CS was detected by the IF assay. **E** UOK109 cells were co-transfected with lentivirus shRNA (NC) or shRNA (NMRK2) and MDH2-Flag, and cultured in the setting of glucose deprivation for 24 h. The binding between MDH2, FH, and CS was detected by IP assay. **F** UOK109 cells were transfected with lentivirus shRNA (NC), shRNA (NMRK2), or shRNA (NMRK2 + GAS5) and cultured in the setting of glucose deprivation for 24 h. The mitochondrial respiration of cells in each group was measured by the Seahorse XF Extracellular Flux Analyzer with a Seahorse XF Cell Mito Stress Kit. **G** Schematic overview of the mechanism concerning lncRNA like NMRK2 mRNA and lnc-GAS5. Data are presented as the mean ± SEM. **P < 0.01

The lnc-GAS5 was confirmed to prevent the formation of the FH-MDH2-CS complex and downregulate the TCA cycle by translocation from cytoplasm to mitochondria after glucose deprivation [38]. In the present study, the result of RIP demonstrated that lncRNA like NMRK2 mRNA could bind to lnc-GAS5 in the setting of glucose deprivation in UOK109 cells (Fig. 6A). lncRNA like NMRK2 mRNA was confirmed to be located simultaneously in mitochondria and cytoplasm (Fig. 6B) and make lnc-GAS5 being detained in the cytoplasm after treatment of glucose deprivation, which may suppress the translocation of lnc-GAS5 to mitochondria, resulting to dissociate the FH-MDH2-CS complex (Fig. 6C, sFig. 5A). In fact, the knockdown of NMRK2 in UOK109 did lead to the reduction in MDH2 binding to CS and FH after glucose deprivation (Fig. 6D, E), which was reversed by GAS5 knockdown (sFig. 5B). Also, the respiration flux was impaired when NMRK2 was knocked down during glucose deprivation. Such a phenomenon was reversed after the interference of lnc-GAS5 (Fig. 6F, sFig. 5C). However, the NMRK2-GAS5 axis did not work when glucose was abundant, which suggested that this mechanism could only account for the enhanced mitochondrial respiration during the early stage of *NONO-TFE3* rRCC progression or at the site of tumor vascular deficiency (sFig. 5D-G). In summary, lncRNA like NMRK2 mRNA could promote the reconstruction of the FH-MDH2-CS complex by binding to lnc-GAS5 in response to nutrient change, resulting to facilitate the TCA cycle and mitochondrial respiration in *NONO-TFE3* rRCC (Fig. 6G).

### LncRNA like NMRK2 mRNA promoted the mitochondrial respiration by enhancing the stability of Hsp10

**Fig. 7:**
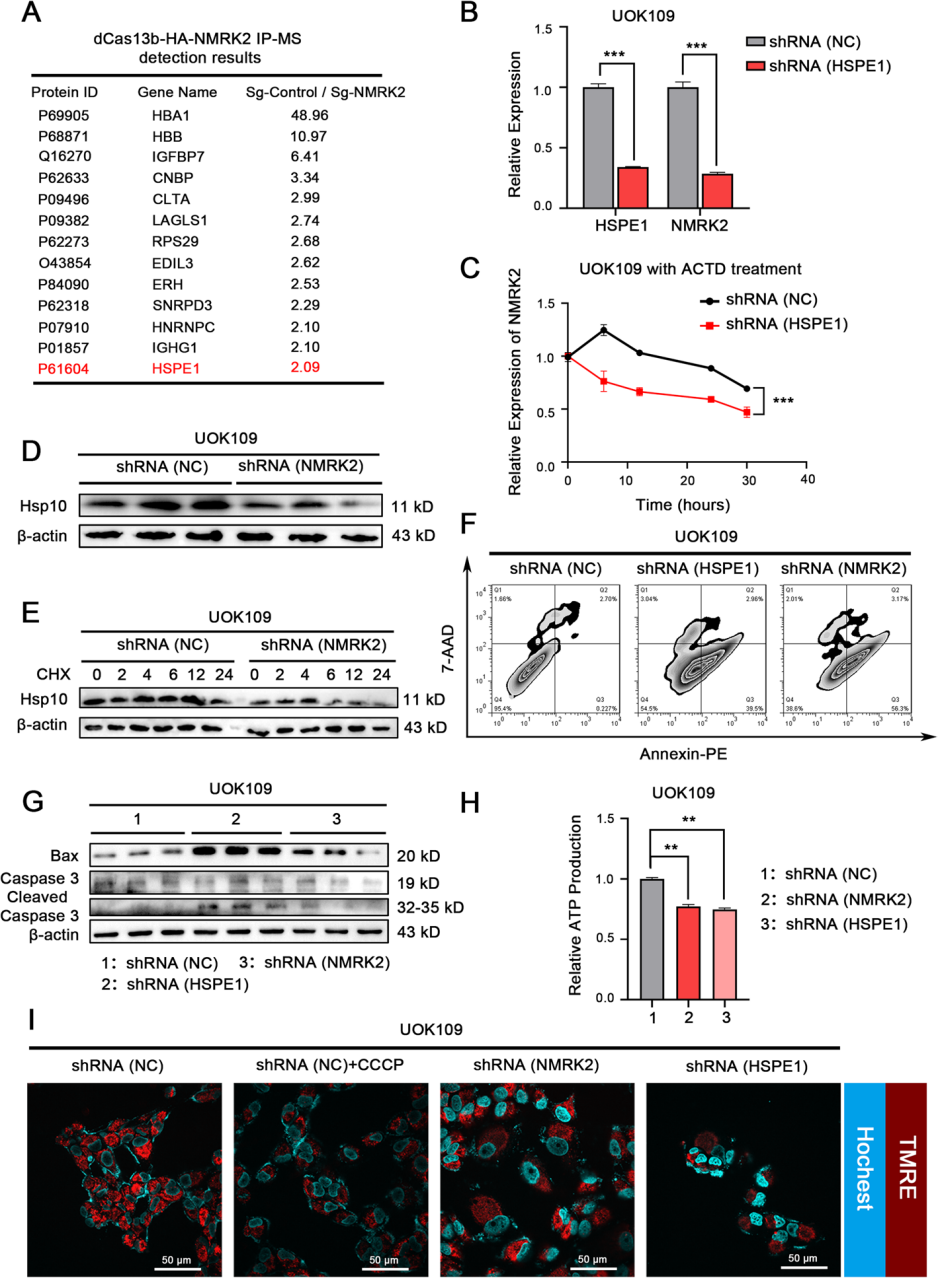
LncRNA like NMRK2 mRNA promoted the mitochondrial respiration of *NONO-TFE3* rRCC by upregulating the protein stability of Hsp10. **A** UOK109 cells were co-transfected with dCas13b-HA and sg-Control/sg-NMRK2, and the NMRK2 binding protein was detected by IP-MS assay. **B** UOK109 cells were transfected with lentivirus shRNA (NC) or shRNA (HSPE1), and the expression of NMRK2 was detected by q-PCR. 18S rRNA was used as the reference gene. **C** UOK109 cells were transfected with lentivirus shRNA (NC) or shRNA (HSPE1) and treated with 10 μM ACTD for 0, 6, 12, 24, and 30 h. The expression of NMRK2 at various time points was detected by q-PCR. 18S rRNA was used as the reference gene. **D** UOK109 cells were transfected with lentivirus shRNA (NC) or shRNA (NMRK2), and the expression of the Hsp10 protein was detected by Western Blotting. β-actin was used as the internal reference. **E** UOK109 cells were transfected with lentivirus shRNA (NC) or shRNA (NMRK2) and treated with 40 μM CHX for 0, 2, 4, 6, 12, and 24 h. The expression of Hsp10 protein at various time points was detected by WB. β-actin was used as the internal reference. **F**, **G** UOK109 cells were transfected with lentivirus shRNA (NC), shRNA (NMRK2), or shRNA (HSPE1), and the apoptosis was assessed by Flow cytometry analysis and Western Blotting. β-actin was used as the internal reference. **H** The ATP production and **I** mitochondrial membrane potential of cells in each group was detected with an Enhanced ATP Assay Kit and a Mitochondrial Membrane Potential Assay Kit. Data are presented as the mean ± SEM. **P < 0.01, ***P < 0.001

To identify the potential binding protein to lncRNA like NMRK2 mRNA, we performed a dcas13b-IP assay followed by label-free mass spectrometry. The result showed that Hsp10, which was involved in mitochondrial unfolded protein response (mtUPR), was verified to bind to NMRK2 (Fig. 7A). The knockdown of HSPE1 which encodes Hsp10 accelerated the degradation of NMRK2 (Fig. 7B, C), while the high expression of NMRK2 in UOK109 promoted the protein stability of Hsp10 (Fig. 7D, E, sFig. 6A). Quantifying the apoptosis assay indicated that the knockdown of NMRK2 or HSPE1 promoted UOK109 cell apoptosis (Fig. 7F, G, sFig. 6B). As mitochondria are the primary source of energy generation, we examined the ATP level of UOK109 cells before or after the silence of NMRK2 or HSPE1. The result indicated that the ATP level was sharply decreased when knocked down the expression of NMRK2 or HSPE1 (Fig. 7H). We also examined the mitochondrial membrane potential (MMP) and found that the MMP was decreased after the knockdown of NMRK2 or HSPE1 in UOK109 cells (F ig. 7I). In summary, lncRNA like NMRK2 mRNA and its binding protein Hsp10 could enhance stability of each other, which activated the mtUPR and promoted the recovery of mitochondrial function.

### The combination of shRNA (NMRK2)-Lentivirus and metformin showed specific and superior anti-tumor activity of *NONO-TFE3* rRCC

**Fig. 8:**
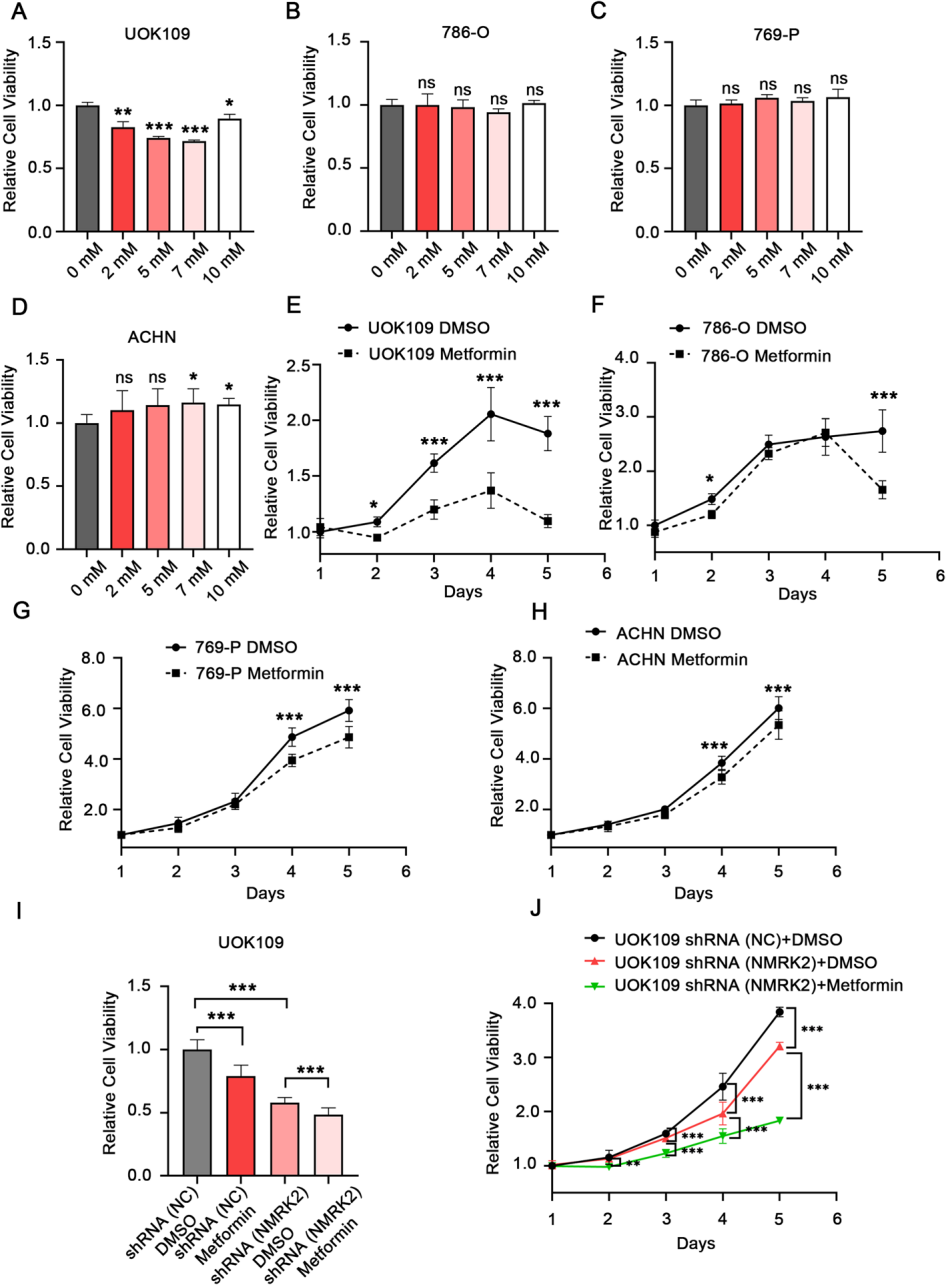
The combination of shRNA (NMRK2) and metformin showed superior efficacy anti-tumor for NONO-TFE3 rRCC. **A**-**D** UOK109, 786-O, 769-P and ACHN were treated with 0 mM, 2 mM, 5 mM, 7 mM, 10 mM metformin for 24 h, respectively. The cell activity of each cell line was detected with CCK-8 kit. **E**–**H** UOK109, 786-O, 769-P and ACHN were treated with 0 mM or 5 mM metformin, and the cell proliferation of each cell line from day 1 to day 5 was tested with CCK-8 kit. **I** UOK109 cells were treated with 0 mM or 5 mM metformin for 24 h after being transfected with shRNA (NC) or shRNA (NMRK2), and the cell activity was detected with CCK-8 kit. **J** UOK109 cells were treated with 0 mM or 5 mM metformin for 5 days after being transfected with shRNA (NC) or shRNA (NMRK2), and the cell proliferation was detected with CCK-8 kit. Data are presented as the mean ± SEM. *P < 0.05, **P < 0.01, ***P < 0.001

Since metformin targets the complex Ι of the electron transport chain, we subsequently investigated the promising clinical applications of shRNA (NMRK2)-lentivirus and metformin in synergistic therapy. Firstly, the specificity of metformin was assessed. Cell activity of *NONO-TFE3* rRCC cell line (UOK109) and ccRCC cell lines (786-O, 769-P and ACHN) was detected with CCK-8 kit after being treated with various concentrations of metformin for 24 h. The results showed that metformin showed cytotoxicity on UOK109 at low concentration (2 mM), while showed no significant cytotoxicity on 786-O, 769-P and ACHN (Fig. 8A-D). The cell proliferation of cells above in 5 days was tested with CCK-8 kit after being treated with 0 mM or 5 mM metformin. The results demonstrated that metformin impaired the proliferation of UOK109 on the second day and showed slight damage effect for 786-O, 769-P and ACHN on day 4 or 5 (Fig. 8E-H). Next, we explore the anti-tumor efficacy of combination of shRNA (NMRK2)-lentivirus and metformin by detecting the cell activity and proliferation of UOK109. The results showed that the combination performed significantly better than either agent alone (Fig. 8I, J). In all, the combination of shRNA (NMRK2)-lentivirus and metformin could be an effective therapeutic strategy for *NONO-TFE3* rRCC.

## Discussion

In this study, we further explored the metabolic features of *TFE3* rRCC and addressed the remaining concerns based on the previous survey. Compared with other subtypes of *TFE3* rRCC, such as *PRCC-TFE3* rRCC, *NONO-TFE3* rRCC had a high level of non-protein-translating NMRK2 mRNA, resulting from a transcriptional-translational conflict process caused by NONO-TFE3 fusion. The up-regulation lncRNA like NMRK2 mRNA improved mitochondrial function of *NONO-TFE3* rRCC in an NAD^+^ kinase-independent manner by three main mechanisms: the interaction between lncRNA like NMRK2 mRNA and miR-26b/181a, lnc-GAS5 or Hsp10 protein. Among the three pathways, lncRNA like NMRK2 mRNA acted as the scaffold for miRNAs, lncRNA, and protein to promote their expression or stability or suppress the function by changing their subcellular localization. For therapeutic application, silencing lncRNA like NMRK2 mRNA could inhibit the cell growth and mitochondrial function of UOK109 cells and suppress the tumor progression of *NONO-TFE3* rRCC.

Recently, a high-throughput sequencing-based study of 79 cases of *TFE3* rRCCs indicated that the TCA cycle and respiratory chain were down-regulated in *TFE3* rRCC compared to adjacent tissues, which is compliant with Warburg Effect [39]. However, it is demonstrated in our previous research that mitochondrial respiration is significantly enhanced in *TFE3* rRCC than that in ccRCC [16]. The above findings suggested that *TFE3* rRCC should be a type of atypical tumor in line with the Warburg Effect, and the mitochondrial respiration could play a crucial role in the progression of *TFE3* rRCC due to the aberrantly active TFE3 fusion protein. Indeed, we verified that interference with the metabolism by targeting mitochondrial respiration is more effective than targeting glycolysis to suppress the progression of *TFE3* rRCC [16]. Unlike most other studies, our research aims to uncover the metabolic differences between *TFE3* rRCC and ccRCC. Our study presents a unique insight into metabolically targeted therapy of *TFE3* rRCC, which could achieve a more precise medical treatment of this tumor.

In addition, *TFE3* rRCC is a class of heterogeneous tumors. Different fusion partners of *TFE3* genes endow the *TFE3* rRCC with distinct molecular signatures. An enhanced RNA regulation network including lncRNA like NMRK2 mRNA and other lncRNA reported promotes the progression of *NONO-TFE3* rRCC in our previous and current studies [31, 35]. This specificity of *NONO-TFE3* rRCC might be caused by the function of RNA editing and modification of the NONO protein [25]. Therefore, the treatment strategy targeting disordered RNAs may be more effective in *NONO-TFE3* rRCC.

We made some novel discoveries during the inquiry into the mechanisms for the role of lncRNA like NMRK2 mRNA in *NONO-TFE3* rRCC. The SLC19A2 which was upregulated by lncRNA like NMRK2 mRNA was first proved to be located in the mitochondrial membrane and function as an NAD^+^ transporter. The up-regulation of SLC19A2 compensated for the kinase function deficiency of NMRK2 protein in *NONO-TFE3* rRCC.

Also, in *NONO-TFE3* rRCC, a unique regulation pathway in response to nutrition deficiency was uncovered, which was different from other tumors. In most tumor models, lnc-GAS5, mainly located in the cytoplasm, is demonstrated to translocate to mitochondria and suppress the TCA cycle by interacting with the MDH2 protein [38]. However, the high expression of lncRNA like NMRK2 mRNA in *NONO-TFE3* rRCC was distributed in both the cytoplasm and mitochondria. Therefore, three scenarios were possible regarding the inhibition of the lnc-GAS5 function under nutrition deficiency observed in the experimental results. First, the lncRNA like NMRK2 mRNA, located in the cytoplasm, could sequester lnc-GAS5 in the cytosol. Second, the lncRNA like NMRK2 mRNA, located in mitochondria, pulled lnc-GAS5 out of the mitochondria. Last, the lncRNA like NMRK2 mRNA which was located in mitochondria could bind with lnc-GAS5 directly and prevent the interaction between lnc-GAS5 and MDH2 protein. Therefore, the up-regulated lncRNA like NMRK2 mRNA could keep the TCA cycle operative in *NONO-TFE3* rRCC.

However, although the mechanism of enhanced mitochondrial respiration regulated by lncRNA like NMRK2 mRNA in *NONO-TFE3* rRCC has been explored thoroughly, there is still some limitations in this study. The lack of spontaneous tumorigenic models and a substantial number of clinical samples are the regrets of this study.

## Conclusion

In summary, we found that lnc-MALAT1 which is transcriptionally up-regulated by NONO-TFE3 fusion protein interrupted the movement of ribosomes on NMRK2 mRNA and led to the translation inhibition of NMRK2 protein. In *NONO-TFE3* rRCC, the high expression of lncRNA like NMRK2 mRNA functioned a vital role in the enhanced mitochondrial respiration in an NAD^+^ kinase-independent manner by promoting the NAD^+^ transport to mitochondria, improving the efficiency of the TCA cycle under low-nutrition setting and upregulating mitochondrial quality control. Inhibition of lncRNA like NMRK2 mRNA could suppress the progression of *NONO-TFE3* rRCC.

### Supplementary Information

**Additional file 1: sFig. 1.** NMRK2 promote the mitochondrial respiration of NONO-TFE3 rRCC. (A) UOK109 cells were transfected with lentivirus shRNA (NC) or shRNA (NMRK2). The ATP production was detected with an ATP assay kit. (B) UOK109 cells were transfected with lentivirus mitochondria-RFP after being transfected with lentivirus shRNA (NC) or shRNA (NMRK2). The number and morphology of mitochondria were observed with confocal microscopy. Data are presented as the mean ± SEM. ***P <0.001.

**Additional file 2: sFig. 2.** Lnc-MALAT1 was upregulated by NONO-TFE3 fusion but not by the PRCC-TFE3 fusion protein. (A) UOK109 cells were transfected with lentivirus shRNA (NC) or shRNA (TFE3). The expression of lnc-MALAT1 was detected by q-PCR. 18S rRNA was used as the reference gene. (B) UOK109 cells were co-transfected with lentivirus shRNA (NC)/shRNA (TFE3) and pGL3-Basic/MALAT1-promoter. The luciferase activity in each group was measured with a Dual-Luciferase Reporter Assay Kit. (C) UOK120 cells were transfected with lentivirus shRNA (NC) or shRNA (TFE3). The expression of lnc-MALAT1 was detected by q-PCR. 18S rRNA was used as the reference gene. (D) UOK120 cells were co-transfected with lentivirus shRNA (NC)/shRNA (TFE3) and pGL3-Basic/MALAT1-promoter. The luciferase activity in each group was measured with a Dual-Luciferase Reporter Assay Kit. Data are presented as the mean ± SEM. *P < 0.05, **P < 0.01, ***P <0.001.

**Additional file 3: sFig. 3.** MiR-26b/miR-181a suppressed the expression of SLC19A2. (A) UOK109 cells were transfected with lentivirus shRNA (NC) or shRNA (NMRK2). The expression of the SLC19A2 protein was detected by Western Blotting. β-actin was used as the internal reference. (B, C) UOK109 cells were transfected with 0, 40, 80, and 150 nM miR-26b/miR-181a mimic RNAs, and the expression of SLC19A2 was detected by q-PCR. 18S rRNA was used as the reference gene. Data are presented as the mean ± SEM. *P < 0.05, **P < 0.01, ***P <0.001.

**Additional file 4: sFig. 4.** SLC19A2 promoted the mitochondrial respiration of NONO-TFE3 rRCC by increasing the NAD+ transportation into mitochondria. (A) UOK109 cells were transfected with lentivirus shRNA (NC), shRNA (NMRK2), or shRNA (SLC19A2). The basal respiration, maximal respiration and spare respiration of cells in each group were assessed and calculated with a Seahorse XF Cell Mito Stress Kit and GraphPad Prism 8. (B) UOK109 cells were transfected with lentivirus shRNA (NMRK2), shRNA (SLC19A2) or shRNA (NMRK2)+pCDH-SLC19A2. Then the cells in each group were treated or untreated with 100 μM NMN for 24 hours. The NAD+ level in mitochondria was measured with an NAD+/NADH Assay Kit. (C, D) UOK109 cells were transfected with lentivirus shRNA (NC), shRNA (NMRK2) or shRNA (SLC19A2). Then the cells in each group were treated or untreated with 100 μM NMN for 24 hours. The basal respiration, maximal respiration and spare respiration of cells in each group were assessed and calculated with a Seahorse XF Cell Mito Stress Kit and GraphPad Prism 8. (E, F) UOK109 cells were transfected with lentivirus shRNA (NC), shRNA (SLC19A2) or shRNA (NMRK2)+pCDH-SLC19A2. Then the cells in each group were treated or untreated with 100 μM NMN for 24 hours. The basal respiration, maximal respiration and spare respiration of cells in each group were assessed and calculated with a Seahorse XF Cell Mito Stress Kit and GraphPad Prism 8. (G) And the ATP production was detected with an ATP assay kit. Data are presented as the mean ± SEM. **P < 0.01, ***P <0.001.

**Additional file 5: sFig. 5.** LncRNA like NMRK2 mRNA promoted the mitochondrial respiration of NONO-TFE3 rRCC by relieving the inhibitory effect of lnc-GAS5 on the TCA cycle. (A) UOK109 cells were transfected with lentivirus shRNA (NC) or shRNA (NMRK2) and cultured in the setting of glucose deprivation for 24 hours. The mitochondria of cells in each group were extracted and purified with a Cell Mitochondria Isolation Kit, and the total RNA in mitochondria was extracted. The expression of lnc-GAS5 was detected by q-PCR. 16S rRNA was used as the reference gene for mitochondrial RNA. (B) UOK109 cells were transfected with lentivirus shRNA (NC), shRNA (NMRK2) or shRNA (NMRK2+GAS5), and cultured in the setting of glucose deprivation for 24 hours. The binding between MDH2, FH, and CS was detected by IP assay. (C) UOK109 cells were transfected with lentivirus shRNA (NC), shRNA (NMRK2), or shRNA (NMRK2+GAS5) and cultured in the setting of glucose deprivation for 24 hours. The basal respiration of cells in each group was assessed and calculated with a Seahorse XF Cell Mito Stress Kit and GraphPad Prism 8. (D, E) UOK109 cells were transfected with lentivirus shRNA (NC), shRNA (NMRK2), or shRNA (NMRK2+GAS5) and cultured in the setting of glucose abundant for 24 hours. The basal respiration, maximal respiration and spare respiration of cells in each group was assessed and calculated with a Seahorse XF Cell Mito Stress Kit and GraphPad Prism 8. (F, G) UOK109 cells were transfected with lentivirus shRNA (NC), shRNA (NMRK2), or shRNA (NMRK2+GAS5) and cultured in the setting of glucose abundant or deprivation for 24 hours. The ATP production of each group was detected with an Enhanced ATP Assay Kit. Data are presented as the mean ± SEM. *P<0.05, **P < 0.01, ***P <0.001.

**Additional file 6: sFig. 6.** LncRNA like NMRK2 mRNA enhanced the mitochondrial respiration of NONO-TEF3 rRCC through promoting the protein stability of Hsp10. (A) UOK109 cells were transfected with lentivirus shRNA (NC) or shRNA (NMRK2), and the expression of the Hsp10 protein was detected by Western Blotting. The quantification results of Western Blotting were calculated by Image J. (B) UOK109 cells were transfected with lentivirus shRNA (NC), shRNA (NMRK2), or shRNA (HSPE1), and the apoptosis was assessed by and Western Blotting. The quantification results of Western Blotting were calculated by Image J. (C) UOK109 cells were transfected with lentivirus shRNA (NC), shRNA (HSPE1), or shRNA (NMRK2)+pCDH-HSPE1, and the ATP production was measured with an Enhanced ATP Assay Kit. (D) UOK109 cells were transfected with lentivirus shRNA (NC), shRNA (NMRK2), shRNA (HSPE1), or shRNA (NMRK2)+pCDH-HSPE1, and the mitochondrial membrane potential of cells in each group was detected with a Mitochondrial Membrane Potential Assay Kit. Data are presented as the mean ± SEM. *P < 0.01, ***P <0.001.

**Additional file 7.**

**Additional file 8.**

## Data Availability

The datasets used and/or analyzed during the current study are available from the corresponding author upon reasonable request.

## Abbreviations

lncRNA: Long non-coding RNA
rRCC: Rearranged renal cell carcinoma
ccRCC: Renal clear cell carcinoma
NT: NONO-TFE3
NM: The protein-coding transcripts
NR: The non-protein-coding transcripts
mtUPR: Mitochondrial unfolded protein response
TFE3: Transcription factor binding to IGHM enhancer 3
NMRK2: Nicotinamide riboside kinase 2
