# Supplementary figures and images for: LncRNA like NMRK2 mRNA functions as a key molecular scaffold to enhance mitochondrial respiration of NONO-TFE3 rearranged renal cell carcinoma in an NAD+ kinase-independent manner
Source: J Exp Clin Cancer Res. 2023 Sep 28;42:252. doi: 10.1186/s13046-023-02837-4 (PMC10537463; doi:10.1186/s13046-023-02837-4)

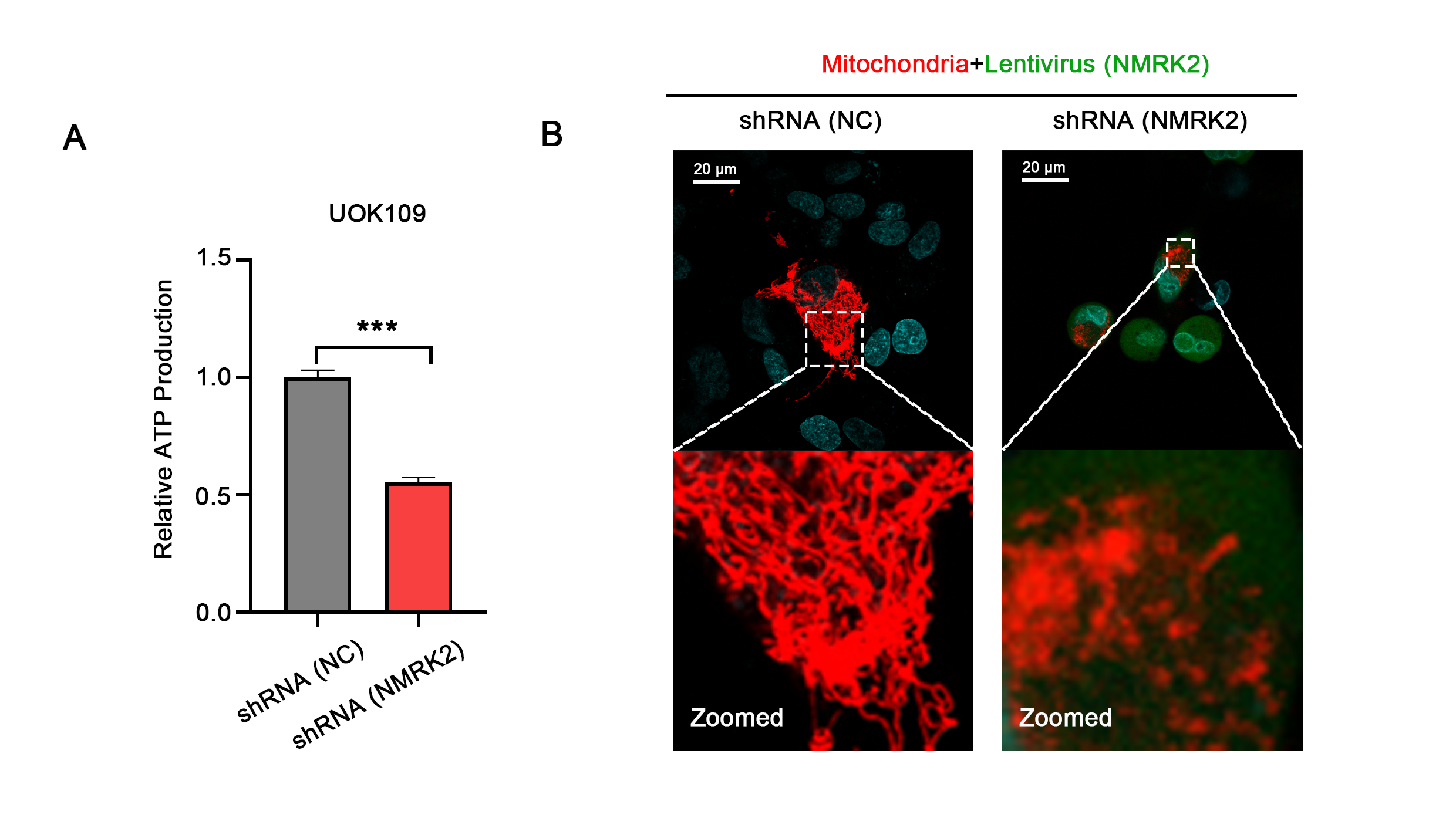

Supplement: Supplementary file 1 — Additional file 1: sFig. 1. NMRK2 promote the mitochondrial respiration of NONO-TFE3 rRCC. (A) UOK109 cells were transfected with lentivirus shRNA (NC) or shRNA (NMRK2). The ATP production was detected with an ATP assay kit. (B) UOK109 cells were transfected with lentivirus mitochondria-RFP after being transfected with lentivirus shRNA (NC) or shRNA (NMRK2). The number and morphology of mitochondria were observed with confocal microscopy. Data are presented as the mean ± SEM. ***P <0.001. [file 13046_2023_2837_MOESM1_ESM.tif]

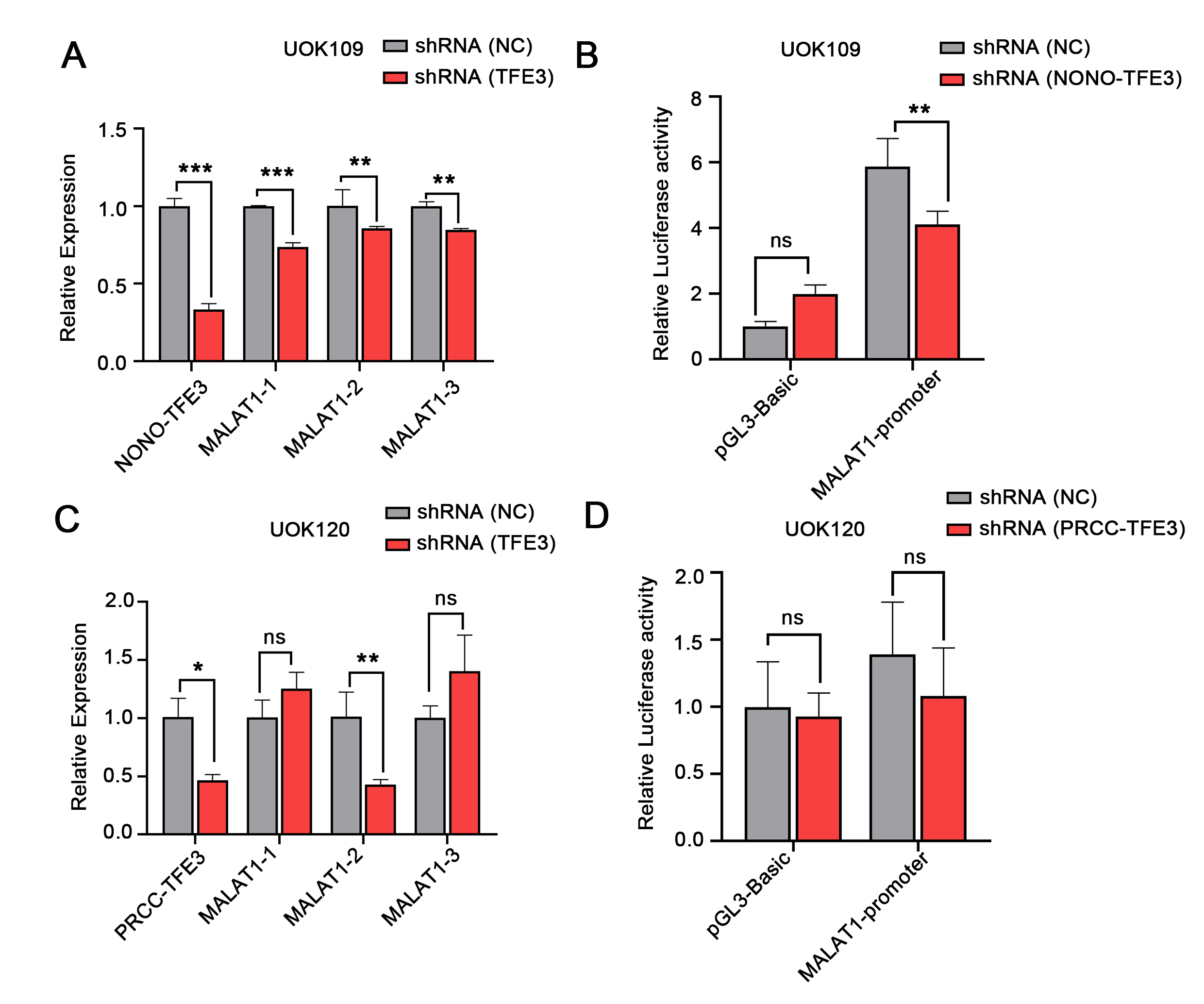

Supplement: Supplementary file 2 — Additional file 2: sFig. 2. Lnc-MALAT1 was upregulated by NONO-TFE3 fusion but not by the PRCC-TFE3 fusion protein. (A) UOK109 cells were transfected with lentivirus shRNA (NC) or shRNA (TFE3). The expression of lnc-MALAT1 was detected by q-PCR. 18S rRNA was used as the reference gene. (B) UOK109 cells were co-transfected with lentivirus shRNA (NC)/shRNA (TFE3) and pGL3-Basic/MALAT1-promoter. The luciferase activity in each group was measured with a Dual-Luciferase Reporter Assay Kit. (C) UOK120 cells were transfected with lentivirus shRNA (NC) or shRNA (TFE3). The expression of lnc-MALAT1 was detected by q-PCR. 18S rRNA was used as the reference gene. (D) UOK120 cells were co-transfected with lentivirus shRNA (NC)/shRNA (TFE3) and pGL3-Basic/MALAT1-promoter. The luciferase activity in each group was measured with a Dual-Luciferase Reporter Assay Kit. Data are presented as the mean ± SEM. *P < 0.05, **P < 0.01, ***P <0.001. [file 13046_2023_2837_MOESM2_ESM.tif]

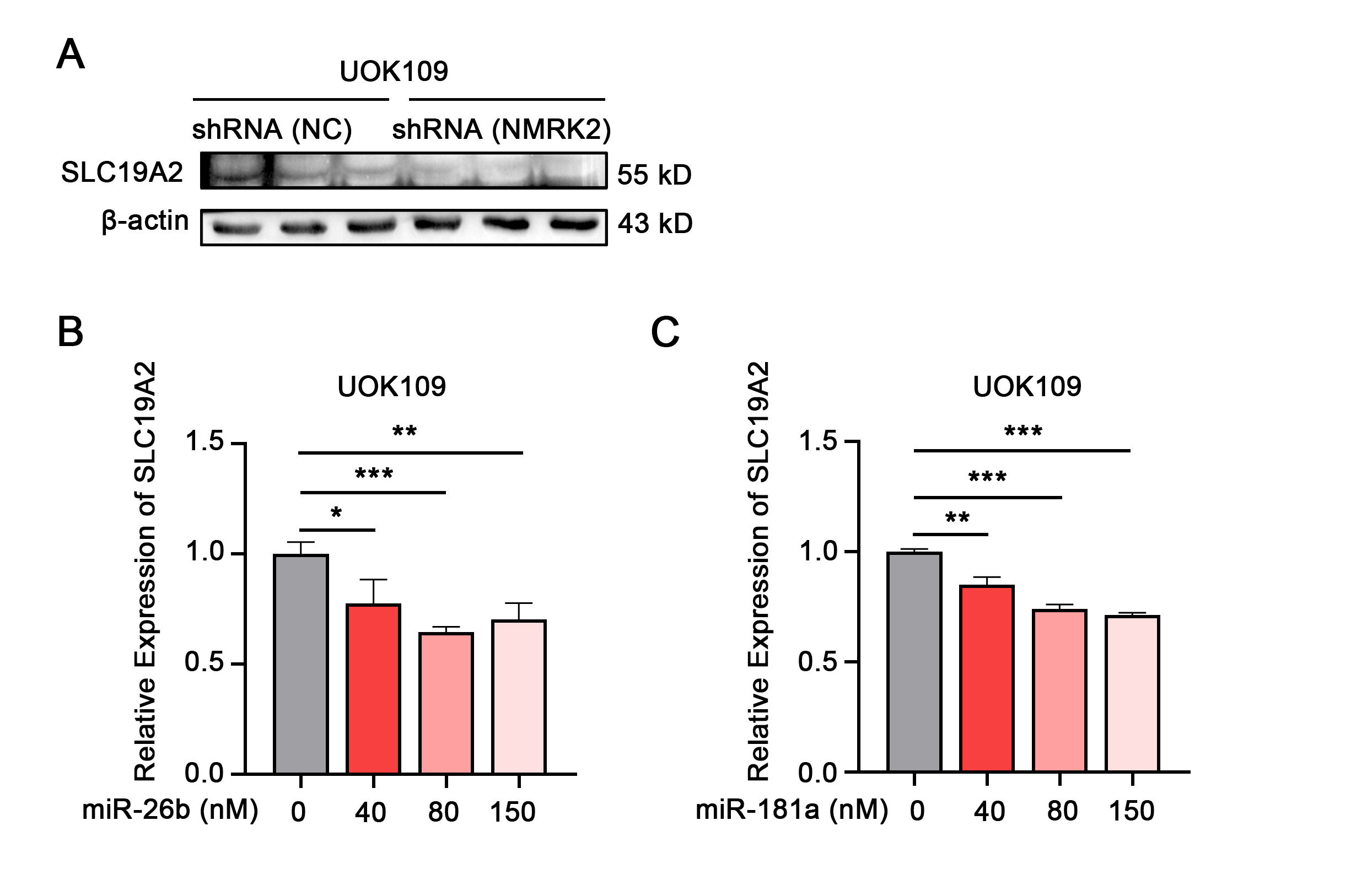

Supplement: Supplementary file 3 — Additional file 3: sFig. 3. MiR-26b/miR-181a suppressed the expression of SLC19A2. (A) UOK109 cells were transfected with lentivirus shRNA (NC) or shRNA (NMRK2). The expression of the SLC19A2 protein was detected by Western Blotting. β-actin was used as the internal reference. (B, C) UOK109 cells were transfected with 0, 40, 80, and 150 nM miR-26b/miR-181a mimic RNAs, and the expression of SLC19A2 was detected by q-PCR. 18S rRNA was used as the reference gene. Data are presented as the mean ± SEM. *P < 0.05, **P < 0.01, ***P <0.001. [file 13046_2023_2837_MOESM3_ESM.tif]

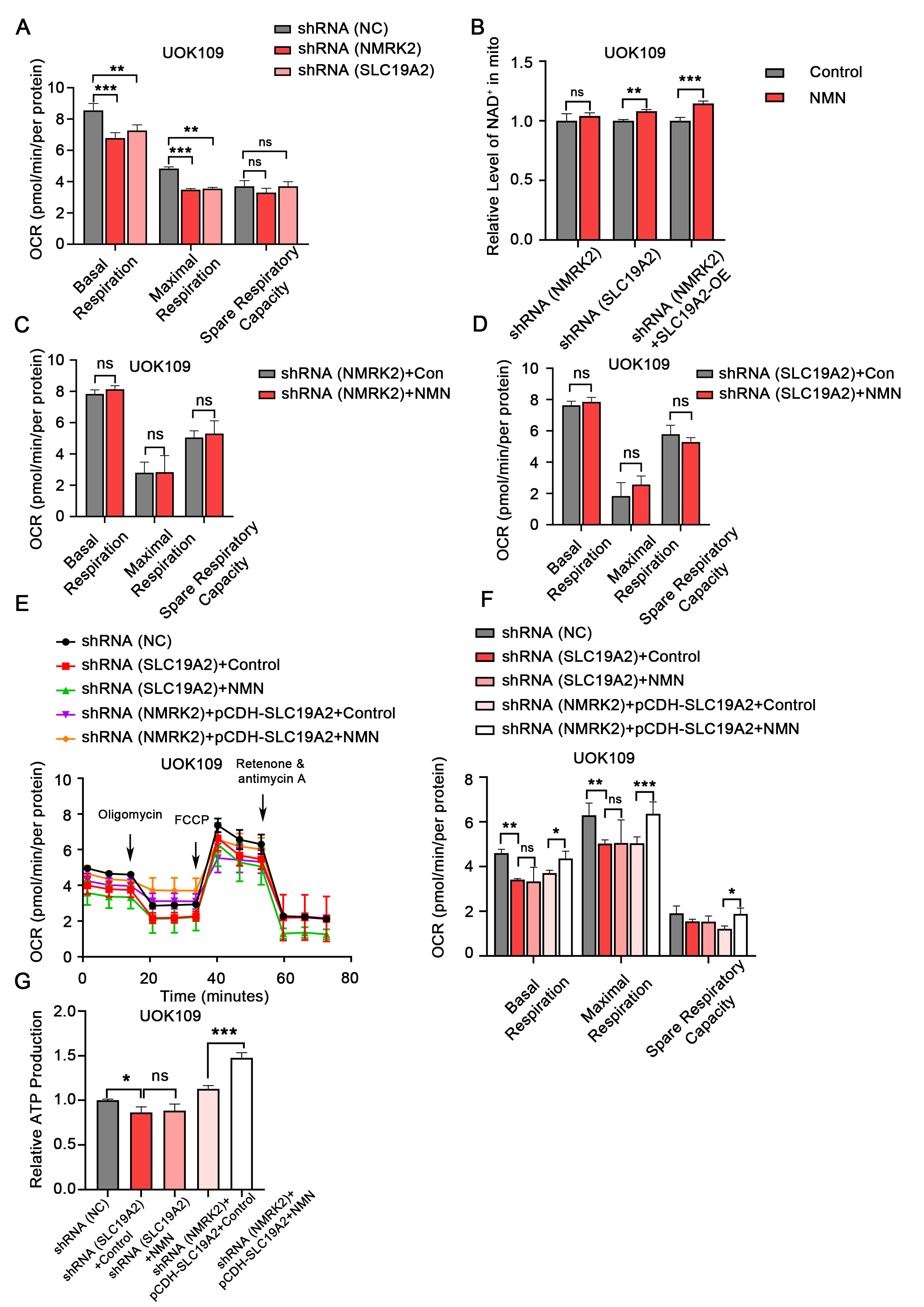

Supplement: Supplementary file 4 — Additional file 4: sFig. 4. SLC19A2 promoted the mitochondrial respiration of NONO-TFE3 rRCC by increasing the NAD+ transportation into mitochondria. (A) UOK109 cells were transfected with lentivirus shRNA (NC), shRNA (NMRK2), or shRNA (SLC19A2). The basal respiration, maximal respiration and spare respiration of cells in each group were assessed and calculated with a Seahorse XF Cell Mito Stress Kit and GraphPad Prism 8. (B) UOK109 cells were transfected with lentivirus shRNA (NMRK2), shRNA (SLC19A2) or shRNA (NMRK2)+pCDH-SLC19A2. Then the cells in each group were treated or untreated with 100 μM NMN for 24 hours. The NAD+ level in mitochondria was measured with an NAD+/NADH Assay Kit. (C, D) UOK109 cells were transfected with lentivirus shRNA (NC), shRNA (NMRK2) or shRNA (SLC19A2). Then the cells in each group were treated or untreated with 100 μM NMN for 24 hours. The basal respiration, maximal respiration and spare respiration of cells in each group were assessed and calculated with a Seahorse XF Cell Mito Stress Kit and GraphPad Prism 8. (E, F) UOK109 cells were transfected with lentivirus shRNA (NC), shRNA (SLC19A2) or shRNA (NMRK2)+pCDH-SLC19A2. Then the cells in each group were treated or untreated with 100 μM NMN for 24 hours. The basal respiration, maximal respiration and spare respiration of cells in each group were assessed and calculated with a Seahorse XF Cell Mito Stress Kit and GraphPad Prism 8. (G) And the ATP production was detected with an ATP assay kit. Data are presented as the mean ± SEM. **P < 0.01, ***P <0.001. [file 13046_2023_2837_MOESM4_ESM.tif]

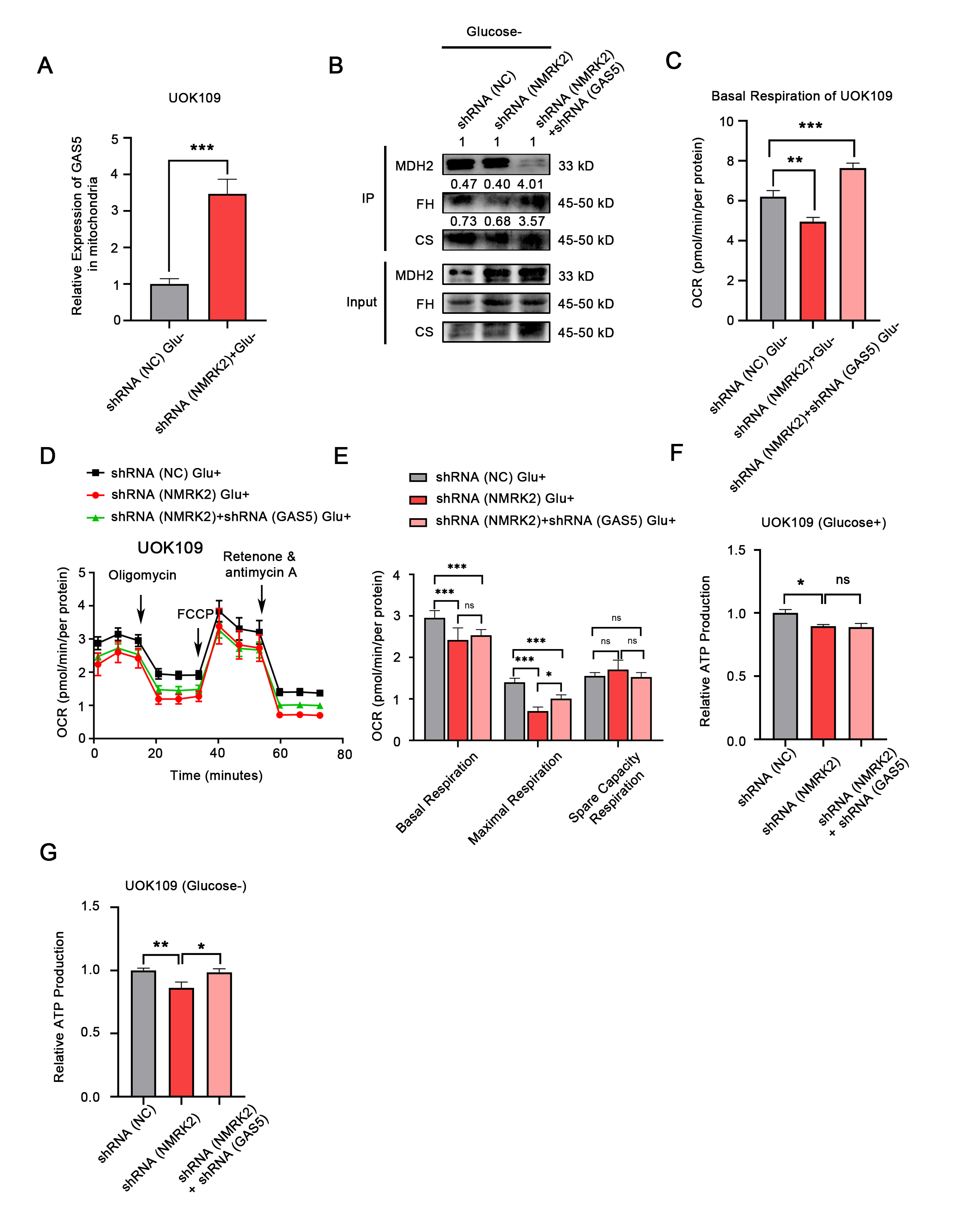

Supplement: Supplementary file 5 — Additional file 5: sFig. 5. LncRNA like NMRK2 mRNA promoted the mitochondrial respiration of NONO-TFE3 rRCC by relieving the inhibitory effect of lnc-GAS5 on the TCA cycle. (A) UOK109 cells were transfected with lentivirus shRNA (NC) or shRNA (NMRK2) and cultured in the setting of glucose deprivation for 24 hours. The mitochondria of cells in each group were extracted and purified with a Cell Mitochondria Isolation Kit, and the total RNA in mitochondria was extracted. The expression of lnc-GAS5 was detected by q-PCR. 16S rRNA was used as the reference gene for mitochondrial RNA. (B) UOK109 cells were transfected with lentivirus shRNA (NC), shRNA (NMRK2) or shRNA (NMRK2+GAS5), and cultured in the setting of glucose deprivation for 24 hours. The binding between MDH2, FH, and CS was detected by IP assay. (C) UOK109 cells were transfected with lentivirus shRNA (NC), shRNA (NMRK2), or shRNA (NMRK2+GAS5) and cultured in the setting of glucose deprivation for 24 hours. The basal respiration of cells in each group was assessed and calculated with a Seahorse XF Cell Mito Stress Kit and GraphPad Prism 8. (D, E) UOK109 cells were transfected with lentivirus shRNA (NC), shRNA (NMRK2), or shRNA (NMRK2+GAS5) and cultured in the setting of glucose abundant for 24 hours. The basal respiration, maximal respiration and spare respiration of cells in each group was assessed and calculated with a Seahorse XF Cell Mito Stress Kit and GraphPad Prism 8. (F, G) UOK109 cells were transfected with lentivirus shRNA (NC), shRNA (NMRK2), or shRNA (NMRK2+GAS5) and cultured in the setting of glucose abundant or deprivation for 24 hours. The ATP production of each group was detected with an Enhanced ATP Assay Kit. Data are presented as the mean ± SEM. *P<0.05, **P < 0.01, ***P <0.001. [file 13046_2023_2837_MOESM5_ESM.tif]

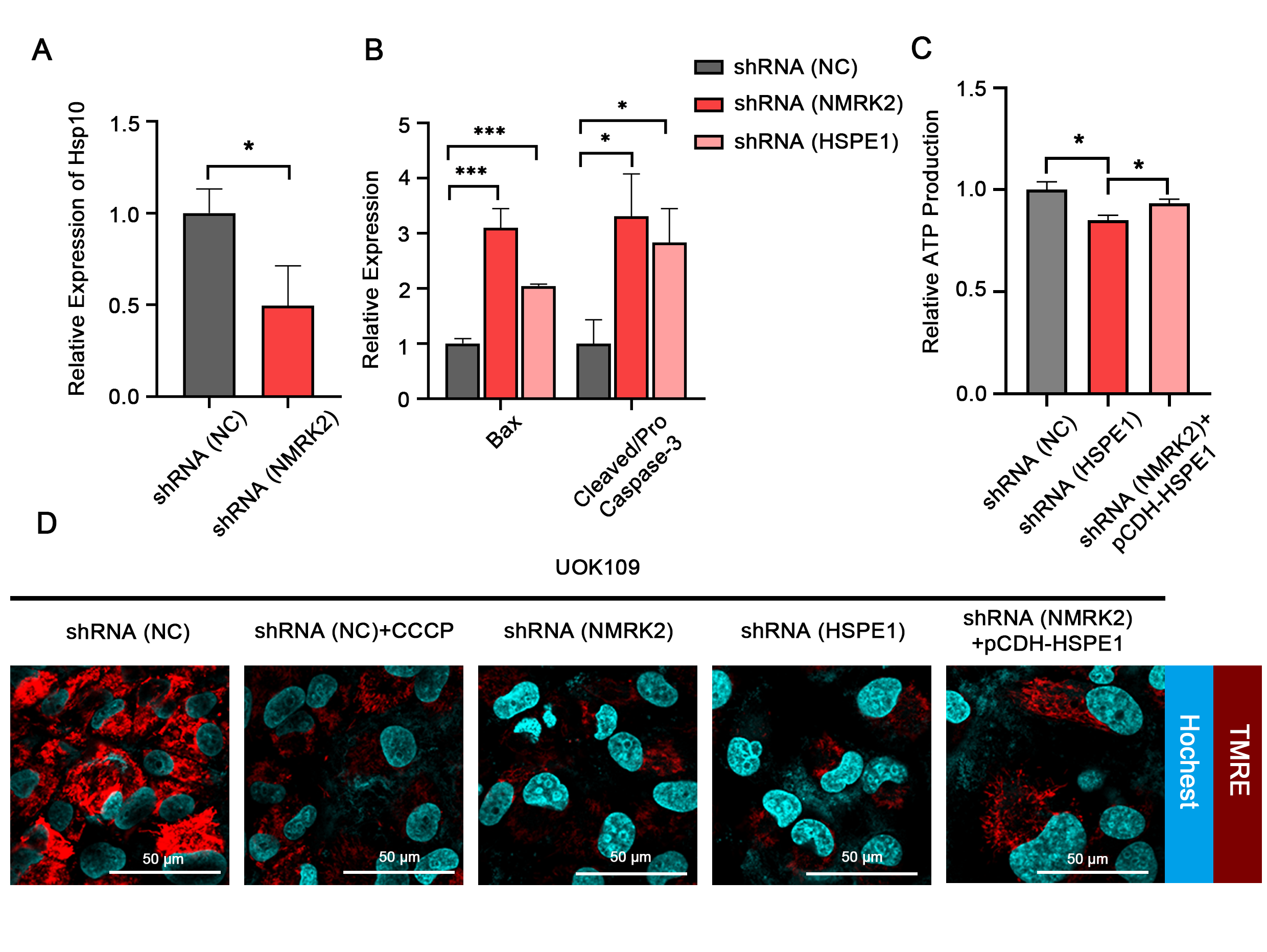

Supplement: Supplementary file 6 — Additional file 6: sFig. 6. LncRNA like NMRK2 mRNA enhanced the mitochondrial respiration of NONO-TEF3 rRCC through promoting the protein stability of Hsp10. (A) UOK109 cells were transfected with lentivirus shRNA (NC) or shRNA (NMRK2), and the expression of the Hsp10 protein was detected by Western Blotting. The quantification results of Western Blotting were calculated by Image J. (B) UOK109 cells were transfected with lentivirus shRNA (NC), shRNA (NMRK2), or shRNA (HSPE1), and the apoptosis was assessed by and Western Blotting. The quantification results of Western Blotting were calculated by Image J. (C) UOK109 cells were transfected with lentivirus shRNA (NC), shRNA (HSPE1), or shRNA (NMRK2)+pCDH-HSPE1, and the ATP production was measured with an Enhanced ATP Assay Kit. (D) UOK109 cells were transfected with lentivirus shRNA (NC), shRNA (NMRK2), shRNA (HSPE1), or shRNA (NMRK2)+pCDH-HSPE1, and the mitochondrial membrane potential of cells in each group was detected with a Mitochondrial Membrane Potential Assay Kit. Data are presented as the mean ± SEM. *P < 0.01, ***P <0.001. [file 13046_2023_2837_MOESM6_ESM.tif]
