# Supplementary material for: LncRNA like NMRK2 mRNA functions as a key molecular scaffold to enhance mitochondrial respiration of NONO-TFE3 rearranged renal cell carcinoma in an NAD+ kinase-independent manner
Source: J Exp Clin Cancer Res. 2023 Sep 28;42:252. doi: 10.1186/s13046-023-02837-4 (PMC10537463; doi:10.1186/s13046-023-02837-4)
Supplement: Supplementary file 7 — Additional file 7. [file 13046_2023_2837_MOESM7_ESM.docx]

**Long non-coding RNA like mRNA NMRK2 functions as a key molecular scaffold to enhance mitochondrial respiration through NAD^+^ kinase-independent manner in *NONO-TFE3* rearranged renal cell carcinoma**

Yi Chen^1,2,6^, Yanwen Lu^3,6^, Lei Yang^4,6^, Wenliang Ma^3^, Yuhan Dong^1,2^, Shuoming Zhou^3^, Ning Liu^5*^, Weidong Gan^3*^, Dongmei Li^1,2*^

1. Immunology and Reproduction Biology Laboratory & State Key Laboratory of Analytical Chemistry for Life Science, Medical School, Nanjing University, Nanjing, Jiangsu 210093, China

2. Jiangsu Key Laboratory of Molecular Medicine, Nanjing University, Nanjing, Jiangsu 210093, China

3. Department of Urology, Affiliated Drum Tower Hospital of Medical School of Nanjing University, Nanjing, Jiangsu 210008, China.

4. Department of Clinical Biobank & Institute of Oncology, Affiliated Hospital of Nantong University, Nantong Jiangsu 226000, China.

5. Department of Urology, Nanjing First Hospital, Nanjing Medical University, Nanjing, Jiangsu 210001, China.

6. These authors contributed equally.

* Corresponding Author:

Dongmei Li, Immunology and Reproduction Biology Laboratory & State Key Laboratory of Analytical Chemistry for Life Science, Medical School, Nanjing University, Nanjing, Jiangsu 210093, China

Email: lidm@nju.edu.cn

Weidong Gan, Department of Urology, Affiliated Drum Tower Hospital of Medical School of Nanjing University, Nanjing, Jiangsu 210008, China

Email: gwd@nju.edu.cn

Ning Liu: Department of Urology, Nanjing First Hospital, Nanjing Medical University, Nanjing, Jiangsu 210001, China

Email: LNnjubg@163.com

**Key Resource Table**

| REAGENT or RESOURCE | SOURCE | IDENTIFIER |
| --- | --- | --- |
| **Antibodies** | | |
| Ki67 | Proteintech | 27309-1-AP |
| TFE3 | Abcam | ab93808 |
| NMRK2 | Sigma | PA5-24607 |
| Flag | Proteintech | 20543-1-AP |
| β-actin | Proteintech | 66009-1-Ig |
| M7G | Synaptic System | 201221 |
| HA | Abcam | ab9110 |
| SLC19A2 | Abcam | ab229680 |
| Tom20 | Proteintech | 66777-1-Ig |
| GAPDH | Proteintech | AC033 |
| MDH2 | Proteintech | 15462-1-AP |
| CS | Proteintech | 16131-1-AP |
| FH | Proteintech | 11375-1-AP |
| Hsp10 | Abclonal | A5580 |
| Bax | Proteintech | 50599-2-Ig |
| Caspase 3 | Proteintech | 19677-1-AP |
| Cleaved Caspase 3 | CST | D175 |
| Argonaute 2 | CST | C34C6 |
| HRP- conjugated goat anti-mouse secondary antibody | Cell Signaling Technology | 7074 |
| HRP- conjugated goat antirabbit secondary antibody | Boster | BA1050 |
| **Chemicals** | | |
| Glucose | Agilent Technologies | 103577-100 |
| L-glutamine | Beyotime | C0212 |
| Pyruvate | Agilent Technologies | 103578-100 |
| Seahorse XF DMEM | Agilent Technologies | 100840-000 |
| Opti-MEM | Gibco | 31985-070 |
| DMEM (High Glucose) | Wisent | 319006028 |
| DMEM (Without Glucose) | Wisent | 319061010 |
| Fetal Bovine Serum | Celligent | CG0430B |
| Penicillin/Streptomycin | Wisent | 450-201-EL |
| Trypsin/EDTA | Wisent | 325-043-EL |
| Puromycin Dihydrochloride | Beyotime | ST551 |
| Cut Smart Buffer | Biolabs | 10085425 |
| 2xUniversal Ligation Mix | Vazyme | C311-01-AA |
| 2xRapid Taq Master Mix | Vazyme | P222-01 |
| 2xPhanta Flash Master Mix | Vazyme | P520-02 |
| Ampicillin, Solid Salt | Solarbio | 69-52-3 |
| HiscriptQ RT Super Mix for q-PCR | Vazyme | R122-01 |
| Taq Pro Universal SYBR q-PCR Master Mix | Vazyme | Q712-02 |
| RNase-free H_2_O | Fcmacs | FMS-WB034 |
| RNA Easy Isolation Reagent | Vazyme | R701-02-AA |
| Anti-Flag Affinity Gel | MCE | 112728 |
| Anti-HA Magnetic Beads | MCE | HY-K0201 |
| Antifade Mounting Medium with DAPI | Beyotime | P0131 |
| β-Nicotinamide mononucleotide | MCE | HY-F0004 |
| Cycloheximide | MCE | HY-12320 |
| Actinomycin D | MCE | HY-17559 |
| RIPA | Beyotime | P0013C |
| Matrigel | Corning | 356234 |
| LipoFiter 3.0 | Hanbio | HB-LF-1000 |
| Polybrene | Solarbio | H8761 |
| Phosphatase inhibitor cocktail | MCE | HY-K0022 |
| Protease inhibitor cocktail | MCE | HYK0010 |
| **Critical Commercial Assays** | | |
| Cell Counting Kit | Yeasen | C8216960 |
| [Seahorse XF Cell Mito Stress Test Kit](https://www.agilent.com.cn/cs/library/flyers/public/5991-7118EN.pdf) | Agilent Technologies | 103015-100 |
| Enhanced ATP Assay Kit | Beyotime | S0027 |
| IHC Kit | Servicebio | G1215 |
| RNA FISH Kit | Gene Pharma | F11202/F21202 |
| RIP Kit | Millipore | 17-701 |
| EndoFree Mini Plasmid Kit | TianGen | DP118-02 |
| Ribosome Extraction Kit | BJBALB | HR8197 |
| Dual-Luciferase Reporter Assay Kit | Vazyme | PL101-01 |
| NAD+/NADH Assay Kit with WST-8 | Beyotime | S0175 |
| Cell Mitochondria Isolation Kit | Beyotime | C3601 |
| Annexin V-PE/7-AAD Apoptosis Detection Kit | Vazyme | A213-01/02 |
| Mitochondrial Membrane Potential Assay Kit with TMRE | Beyotime | C2001S |
| StarPrep Gel Extraction Kit | GeneStar | D205-04 |
| ClonExpress Ⅱ One-Step Cloning Kit | Vazyme | C112-02 |
| Mut Express Ⅱ Fast Mutagenesis Kit | Vazyme | C214-01 |
| FDbio-Femto ECL Kit | Fdbio Science | FD8030 |
| BCA Protein Quantification Kit | Yeasen | 20201ES76 |
| HiScript 1st Strand cDNA Synthesis Kit | Vazyme | R111-01/02 |
| SYBR Green Q-PCR Kit | Vazyme | Q711-02 |
| **Experimental Models: Cell Lines** | | |
| UOK109 | gifts of Dr. W. Marston Linehan, National Cancer Institute,  Bethesda, MD | N/A |
| UOK120 | gifts of Dr. W. Marston Linehan, National Cancer Institute,  Bethesda, MD | N/A |
| 786-O | ATCC | CRL1932 |
| HEK293T | ATCC | CRL3216 |
| HK-2 | ATCC | CRL2190 |
| ACHN | ATCC | CRL1611 |
| 769-P | ATCC | CRL1933 |
| **Experimental Models: Plasmids** | | |
| pLV-shRNA | KeLei | KL-ZL-1225 |
| pCDH-DsRed | N/A | N/A |
| psPAX2 | N/A | N/A |
| pMD2.G | N/A | N/A |
| pcDNA3.1-3xFlag | Youbao | VT-8001 |
| EF1a-dPSPCas13b-NES-HIV | Miaoling Bio | P8305 |
| pBiFC-VC155 | Miaoling Bio | P0680 |
| pBiFC-VN173 | Miaoling Bio | P0681 |
| pmiRGLO | Youbao | VT-1439 |
| [pLV-mitoDsRed](http://www.miaolingbio.com/plasmid/P21276.html) | Miaoling Bio | P21276 |
| pRL-TK | Youbao | VT-1568 |
| **Experimental Models: Escherichia coli strains** | | |
| Stbl3 | NCM Biotech | MC012 |
| DH5α | Vazyme | C502-02 |
| **Experimental Models: Animals** | | |
| BALB/c nude mice | Gempharmatech | D000521 |
| **Oligonucleotides** | | |
| NMRK2 probe | Gempharmatech | N/A |
| GAS5 probe | Gempharmatech | N/A |
| U6 probe | Gempharmatech | N/A |
| 18S probe | Gempharmatech | N/A |
| miR26b mimic RNA | Gempharmatech | N/A |
| miR181a mimic RNA | Gempharmatech | N/A |
| Negative control RNA | Gempharmatech | N/A |
| NMRK2 shRNA | Tsingke Biotechnology | N/A |
| MALAT1 shRNA | Tsingke Biotechnology | N/A |
| SLC19A2 shRNA | Tsingke Biotechnology | N/A |
| HSPE1 shRNA | Tsingke Biotechnology | N/A |
| **Software and website** | | |
| GraphPad Prism 8 | GraphPad Software, Inc | N/A |
| Image J | National Institutes of Health | N/A |
| Seahorse Wave | Agilent Technologies | N/A |
| Snapgene | Dotmatics | N/A |
| Flowjo 7.6 | Acresso | N/A |
| CE Design | Vazyme | N/A |
| miRNA Design | Vazyme | N/A |
| miRDB | https://mirdb.org/ | N/A |
| RNA Inter | http://www.rna-society.org/ | N/A |
| miRbase | https://www.mirbase.org/ | N/A |
| Sigma | https://www.sigmaaldrich.cn/CN/ | N/A |
| NCBI | https://www.ncbi.nlm.nih.gov/ | N/A |
