# Supplementary material for: LncRNA like NMRK2 mRNA functions as a key molecular scaffold to enhance mitochondrial respiration of NONO-TFE3 rearranged renal cell carcinoma in an NAD+ kinase-independent manner
Source: J Exp Clin Cancer Res. 2023 Sep 28;42:252. doi: 10.1186/s13046-023-02837-4 (PMC10537463; doi:10.1186/s13046-023-02837-4)
Supplement: Supplementary file 8 — Additional file 8. [file 13046_2023_2837_MOESM8_ESM.docx]

**Long non-coding RNA like mRNA NMRK2 functions as a key molecular scaffold to enhance mitochondrial respiration through NAD^+^ kinase-independent manner in *NONO-TFE3* rearranged renal cell carcinoma**

Yi Chen^1,2,6^, Yanwen Lu^3,6^, Lei Yang^4,6^, Wenliang Ma^3^, Yuhan Dong^1,2^, Shuoming Zhou^3^, Ning Liu^5*^, Weidong Gan^3*^, Dongmei Li^1,2*^

1. Immunology and Reproduction Biology Laboratory & State Key Laboratory of Analytical Chemistry for Life Science, Medical School, Nanjing University, Nanjing, Jiangsu 210093, China

2. Jiangsu Key Laboratory of Molecular Medicine, Nanjing University, Nanjing, Jiangsu 210093, China

3. Department of Urology, Affiliated Drum Tower Hospital of Medical School of Nanjing University, Nanjing, Jiangsu 210008, China.

4. Department of Clinical Biobank & Institute of Oncology, Affiliated Hospital of Nantong University, Nantong Jiangsu 226000, China.

5. Department of Urology, Nanjing First Hospital, Nanjing Medical University, Nanjing, Jiangsu 210001, China.

6. These authors contributed equally.

* Corresponding Author:

Dongmei Li, Immunology and Reproduction Biology Laboratory & State Key Laboratory of Analytical Chemistry for Life Science, Medical School, Nanjing University, Nanjing, Jiangsu 210093, China

Email: lidm@nju.edu.cn

Weidong Gan, Department of Urology, Affiliated Drum Tower Hospital of Medical School of Nanjing University, Nanjing, Jiangsu 210008, China

Email: gwd@nju.edu.cn

Ning Liu: Department of Urology, Nanjing First Hospital, Nanjing Medical University, Nanjing, Jiangsu 210001, China

Email: LNnjubg@163.com

**Primers used for plasmids construction.**

| Target  gene | Primer sequence (5’-3’) | |
| --- | --- | --- |
|  | Forward | Reverse |
| NONO-TFE3-Flag | gggagacccaagctggctagcATGCAGAGTAATAAAACTTTTAACTTGGA | tccttgtagtcacttaagctGGACTCCTCTTCCATGCTGAAG |
| NONO (1-9 Exon)-Flag | gggagacccaagctggctagcgccaccATGCAGAGTAATAAAACTTTTAACTTGGA | tccttgtagtcacttaagctCGCATCAGGGAAGGTTCCC |
| TFE3 (6-10 Exon)-Flag | gggagacccaagctggctagcgccaccATGCTGCCTGTGTCAGGGAA | tccttgtagtcacttaagctGGACTCCTCTTCCATGCTGAAG |
| V_N_-MDH2 | tgctggatatctgcagaattATGCTCTCCGCCCTCGCC | cttggtaccgagctcggatccCTTCAGGGTCTTCACGAAATCTTC |
| V_C_-FH | tggccatggaggcccgaattATGTACCGAGCACTTCGGCTC | gacgggtacctcgagagatctaCTTTGGACCCAGCATGTCCTT |
| V_C_-CS | tggccatggaggcccgaattATGGCTTTACTTACTGCGGCC | gacgggtacctcgagagatctaCCCTGACTTAGAGTCCACAAACTTC |
| MALAT1-promoter | cgagctcttacgcgtgctagcCTCCCTCATTAATACAATATAAAAATTATTTAC | cagtaccggaatgccaagcttCCGGGCTTCTGCGTTGCT |
| SLC19A2-3’UTR (miR-26b) | tgtttaaacgagctcgctagcTAGATCATCTTGAATATTTGCTTAAAATTG | tgcctgcaggtcgactctagaAGCAGCCCCTTGGTAGACTGA |
| SLC19A2-3’UTR (miR-181a-1) | tgtttaaacgagctcgctagcACAGCAACTGCCTGGATGTGT | tgcctgcaggtcgactctagaTTCCCGGAACACAAGGTATTAGTC |
| SLC19A2-3’UTR (miR-181a-2) | tgtttaaacgagctcgctagcAGGTGTACGTATCATTTCTAATTTTAAGTTA | tgcctgcaggtcgactctagaACTTCTAAACAAATTTTACAGAAATAAATAATC |
| Mutated SLC19A2-3’UTR (miR-26b) | CCAAGTgcaggtccAAAATGTTCTATATGCATTTAATTCTGAAA | TTTTggacctgcACTTGGTAATGAAATCACTAGCAATTTT |
| Deleted SLC19A2-3’UTR (miR-26b) | GTGATTTCATTACCAAGTAAAATGTTCTATATGCATTTAATTCTGAAA | ACTTGGTAATGAAATCACTAGCAATTTTAAGCA |
| Mutated SLC19A2-3’UTR (miR-181a-1) | TTAgtccgtgGCATTTCTTGACTTCACAGCAGC | GAAATGCcacggacTAAATATATGTCTACGCTTTAAAAAATCAAA |
| Deleted SLC19A2-3’UTR (miR-181a-1) | AGCATTTCTTGACTTCACAGCAGCCACTTGACT | GTGAAGTCAAGAAATGCTAAATATATGTCTACGCTTTAAAAAATCAAA |
| Mutated SLC19A2-3’UTR (miR-181a-2) | TCATAgtccgtgTCTTGGTTCCCATTGTGACGA | CAAGAcacggacTATGATGAATTTTTTTAAAATAACTTAAAATTAG |
| Deleted SLC19A2-3’UTR (miR-181a-2) | CATCATATCTTGGTTCCCATTGTGACGATTATT | GGGAACCAAGATATGATGAATTTTTTTAAAATAACTTAAAATTAG |
| MDH2-Flag | tgctggatatctgcagaattATGCTCTCCGCCCTCGCC | cttggtaccgagctcggatccCTTCAGGGTCTTCACGAAATCTTC |
| shRNA (NMRK2) | GATCCCATGTACCAGAAGTATAGGCACTCGAGTGCCTATACTTCTGGTACATGTTTTTG | AATTCAAAAACATGTACCAGAAGTATAGGCACTCGAGTGCCTATACTTCTGGTACATGG |
| shRNA (MALAT1)-1 | GATCCGCAGCCCGAGACTTCTGTAAACTCGAGTTTACAGAAGTCTCGGGCTGCTTTTTG | AATTCAAAAAGCAGCCCGAGACTTCTGTAAACTCGAGTTTACAGAAGTCTCGGGCTGCG |
| shRNA (MALAT1)-2 | GATCCGCCCGAGACTTCTGTAAAGGACTCGAGTCCTTTACAGAAGTCTCGGGCTTTTTG | AATTCAAAAAGCCCGAGACTTCTGTAAAGGACTCGAGTCCTTTACAGAAGTCTCGGGCG |
| shRNA (MALAT1)-3 | GATCCGCTCTAAATTGTTGTGGTTCTCTCGAGAGAACCACAACAATTTAGAGCTTTTTG | AATTCAAAAAGCTCTAAATTGTTGTGGTTCTCTCGAGAGAACCACAACAATTTAGAGCG |
| shRNA (HSPE1) | GATCCTAGCGTGAAAGTTGGAGATAACTCGAGTTATCTCCAACTTTCACGCTATTTTTG | AATTCAAAAATAGCGTGAAAGTTGGAGATAACTCGAGTTATCTCCAACTTTCACGCTAG |
| shRNA (SLC19A2) | GATCCCCCTAGTATTTGGTGTAAATACTCGAGTATTTACACCAAATACTAGGGTTTTTG | AATTCAAAAACCCTAGTATTTGGTGTAAATACTCGAGTATTTACACCAAATACTAGGGG |
| shRNA (GAS5) | GATCCGCAGACCTGTTATCCTAAACTCTCGAGAGTTTAGGATAACAGGTCTGCTTTTTG | AATTCAAAAAGCAGACCTGTTATCCTAAACTCTCGAGAGTTTAGGATAACAGGTCTGCG |
| pCDH-SLC19A2 | atagaagattctagagctagcATGGATGTGCCCGGCCCG | atccttcgcggccgcggatccTTATGAAGTGGTTACTTGAGAACTTGATT |
| pCDH-HSPE1 | gacgacaaactcgaggaattcATGGCAGGACAAGCGTTTAGA | ctacccagcggccgcggatccGTCTACGTACTTTCCAAGAATGTCACC |

**Primers used for real-time PCR.**

| Target  gene | Primer sequence (5’-3’) | |  |
| --- | --- | --- | --- |
|  | Forward | Reverse |  |
| 18S rRNA | CAGCCACCCGAGATTGAGCA | TAGTAGCGACGGGCGGTGTG |  |
| TFE3 | TGCCTGTGTCAGGGAATCTG | CGACGCTCAATTAGGTTGTGAT |  |
| NMRK2 | TCAGAACTCGCTGCTGAACC | GAAACGCTCACATGCTGTCC |  |
| NMRK2-NM | GCCTTGGAAGTCGTCCCC | CGTTGGTCATGCCTCCGAT |  |
| NMRK2-NR | GGCCCTGACCTCGTTGGAAAA | TTGGTCATGCCACCCCCG |  |
| GAS5 | ATGGTGGAGTCCAACTTGCC | AGGATAACAGGTCTGCCTGC |  |
| 16S rRNA | TACCCCGCCTGTTTACCA | GGCAGGTCAATTTCACTGG |  |
| MALAT1-1 | AGTACAGCACAGTGCAGCTT | CCCACCAATCCCAACCGTAA |  |
| MALAT1-2 | AGCTCTGTGGTGTGGGATTG | GAGAAGTGGCAAAATGGCGG |  |
| MALAT1-3 | CTGACCCAGGTGCTACACAG | TACCAACCACTCGCTTTCCC |  |
| SLC25A16 | ACAACCCGCAGAGACTTCTAC | ACTCGATCCAATGGAGCAACT |  |
| SLC25A25 | TGACCATCGACTGGAACGAGT | ACATCAAAGATCGTGGAATGCTT |  |
| SLC25A20 | GACACGGTCAAGGTCCGAC | GCAGCCATTCCCCGATATAGC |  |
| SLC19A2 | TTGCCACAGACTACCTCCGT | GCACTTCGACAGTAACTTGTGA |  |
| SLC25A4 | ATCACGCTTGGAGCTTCCTAA | TGCTTCTCAGCACTGATCTGT |  |
| SLC33A1 | TGGTTGATGCGGTCTACGTTA | CAAGCAAACGGTCCACCTG |  |
| SLC25A24 | GGTGCTGTCTCTCGAACAAG | CATCTGTCGAAAGCCACCAAA |  |
| SLC25A37 | GATGGGGACAGCCGAGATG | ACCGGGTACATGACCGAGT |  |

**Probe sequences used for RNA fluorescence in situ hybridization**

| Target transcript | Probe sequence (5’-3’) |
| --- | --- |
| NMRK2 probe | GGAGCGGT+TCAGCACGAGT+TCTAAGAAGTCA+TCCTGATGGA+  TCACGCAGGAGAC+TCCAGCACGTCCCAC+TGTTT |
| GAS5 probe | GTGAGGCAAGACCCTTTCAAGCAGTAA |
| U6 probe | TTTGCGTGTCATCTTCG |
| 18s rRNA probe | CTGCCTTCCTTGGATGTGGGTAGCCGTTTC |

**Primers used for dcas13b-sgRNA systems.**

| Target  RNA | Primer sequence (5’-3’) | |  |
| --- | --- | --- | --- |
|  | Forward | Reverse |  |
| Sg-Con | GATCGAAACGCTCACATGCTGTCCTGC  aagttgtggaaggtccagttttgaggggctattacaactttttt | AATTaaaaaagttgtaatagcccctcaaaactggaccttccacaactt  GCAGGACAGCATGTGAGCGTTTC |  |
| Sg-NMRK2 | GATCccatgtcttcctgggacgaagacaagttgtggaag  gtccagttttgaggggctattacaactttttt | AATTaaaaaagttgtaatagcccctcaaaactggaccttcca  caacttgtcttcgtcccaggaagacatgg |  |
